# Mesoscopic Organization Reveals the Constraints Governing *Caenorhabditis elegans* Nervous System

**DOI:** 10.1371/journal.pone.0009240

**Published:** 2010-02-22

**Authors:** Raj Kumar Pan, Nivedita Chatterjee, Sitabhra Sinha

**Affiliations:** 1 The Institute of Mathematical Sciences, Chennai, Tamil Nadu, India; 2 Vision Research Foundation, Sankara Nethralaya, Chennai, Tamil Nadu, India; University of California, Irvine, United States of America

## Abstract

One of the biggest challenges in biology is to understand how activity at the cellular level of neurons, as a result of their mutual interactions, leads to the observed behavior of an organism responding to a variety of environmental stimuli. Investigating the intermediate or mesoscopic level of organization in the nervous system is a vital step towards understanding how the integration of micro-level dynamics results in macro-level functioning. The coordination of many different co-occurring processes at this level underlies the command and control of overall network activity. In this paper, we have considered the somatic nervous system of the nematode *Caenorhabditis elegans*, for which the entire neuronal connectivity diagram is known. We focus on the organization of the system into modules, i.e., neuronal groups having relatively higher connection density compared to that of the overall network. We show that this mesoscopic feature cannot be explained exclusively in terms of considerations such as, optimizing for resource constraints (viz., total wiring cost) and communication efficiency (i.e., network path length). Even including information about the genetic relatedness of the cells cannot account for the observed modular structure. Comparison with other complex networks designed for efficient transport (of signals or resources) implies that neuronal networks form a distinct class. This suggests that the principal function of the network, viz., processing of sensory information resulting in appropriate motor response, may be playing a vital role in determining the connection topology. Using modular spectral analysis we make explicit the intimate relation between function and structure in the nervous system. This is further brought out by identifying functionally critical neurons purely on the basis of patterns of intra- and inter-modular connections. Our study reveals how the design of the nervous system reflects several constraints, including its key functional role as a processor of information.

## Introduction

The relatively simple nervous systems of invertebrate organisms provide vital insights into how nerve cells integrate sensory information from the environment, resulting in a coordinated response. Analysing the intermediate or mesoscopic level of organization in such systems is a crucial step in understanding how micro-level activity of single neurons and their interactions eventually result in macro-level behavior of the organism [1]. The nematode *Caenorhabditis elegans* is a model organism on which such an analysis can be performed, as its entire neuronal wiring layout has been completely mapped [2]. This information enables one to trace in full the course of activity along the neuronal network, from sensory stimulation to motor response [3]. We study its somatic nervous system, comprising 282 neurons that control all activity except the pharyngeal movements. This can lead to an understanding of the command and control processes occurring at the mesoscopic level that produce specific functional responses, including avoidance behavior and movement along a chemical gradient. The neuron locations as well as their connections being completely determined by the genetic program, are almost invariant across individual organisms [3]. Further, unlike in higher organisms, the connections do not change with time in the adult nematode [4]. In combination with the possibility of experimenting on the role of single neurons in different functional modalities, these invariances allow one to uniquely identify the important neurons in the system having specific behavioral tasks.

The recent developments in the theory of complex graphs has made available many analytical tools for studying biological networks [5], [6]. The initial emphasis was on developing gross macroscopic descriptions of such systems using measures such as average path length between nodes of the network, the clustering among nodes and the distribution of degree (the number of links per node). However, such global characterizations of systems ignore significant local variations in the connection topology that are often functionally important. Therefore, investigating the network at a mesoscopic level which considers the broad patterns in the inhomogeneous distribution of connections, may reveal vital clues about the working of an organism that could be hidden in a global analysis. Further, these large-scale features help in understanding how coordination and integration occur across different parts of the system, in contrast to a study of microscopic patterns comprising only a few neurons, e.g., motifs [7].

The existence of *modules*, marked by the occurrence of groups of densely connected nodes with relatively fewer connections between these groups [8], provides a natural meso-level description of many complex systems [9]. In biological networks, such modular organization has been observed across many length scales, from the intra-cellular protein-protein interaction system [10], [11] to food webs comprising various species populations [12]. The relation between certain modules and specific functions, e.g., in metabolic networks [13], helps us to understand how different functions are coordinated in the integral performance of complex biological networks.

**Figure 1:**
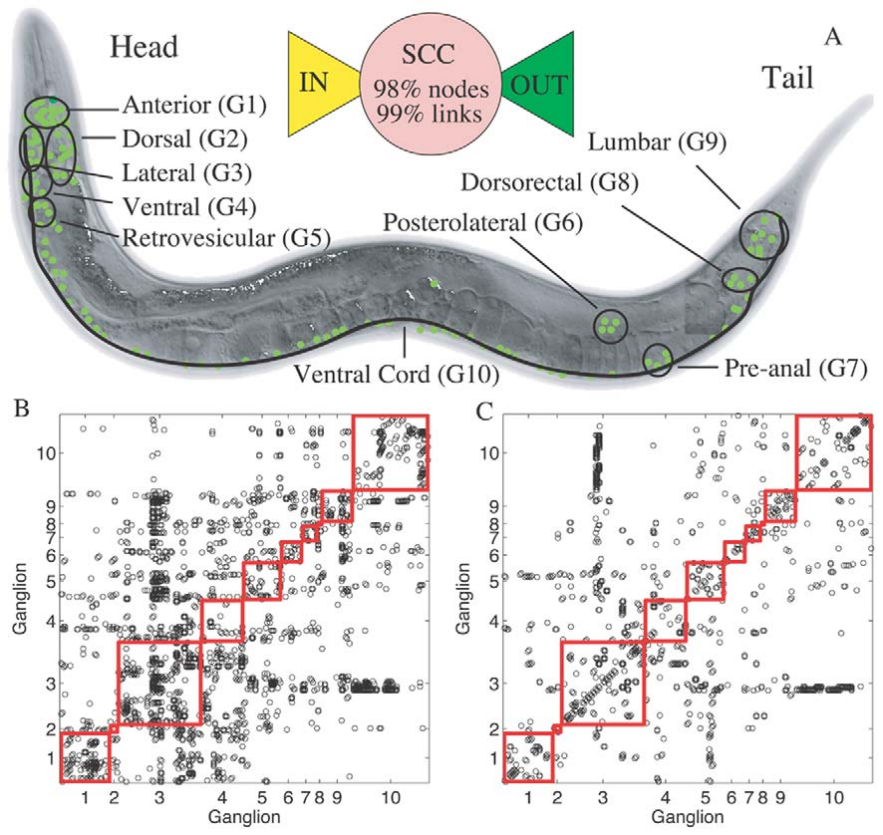
Neuronal position and connectivity in the somatic nervous system of the nematode *C. elegans* indicating the different ganglia. (A) Schematic diagram of *C. elegans*, indicating the different ganglia. (Inset) Schematic representation of connectivity between the neurons, partitioned into a *strongly connected component* (SCC), an *in-component* (IN), and an *out-component* (OUT). A directed path exists from any neuron in IN to any neuron in OUT through neurons in SCC, all of whose members can be reached from each other. The large SCC suggests that it is possible to transfer signals between almost all neurons of the network. The IN and OUT components have only 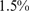 and 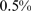, respectively, of the 279 connected neurons in the somatic nervous system. (B, C) The connectivity matrix corresponding to the (B) Synaptic and (C) Gap-junctional connections between the somatic system neurons. In all figures, the partition symbols correspond to (G1) Anterior, (G2) Dorsal, (G3) Lateral, (G4) Ventral, (G5) Retrovesicular, (G6) Posterolateral, (G7) Preanal, (G8) Dorsorectal and (G9) Lumbar ganglion, and (G10) the Ventral cord.

Modular organization in the brains of different species have been observed, both in *functional* networks derived from EEG/MEG and fMRI experiments and in *structural* networks obtained from tracing anatomical connections [14]. Functionally defined networks are constructed by considering brain areas, comprising a large number of localized neuronal groups, which are linked if they are simultaneously active. Such systems have been shown to be modular for both human [15], [16] and non-human [17] subjects. Tract-tracing studies in the brains of cat [18] and macaque [19] have also revealed a modular layout in the structural inter-connections between different cortical areas. However, as neurons are the essential building blocks of the nervous system, ideally one would like to explore the network of interconnections between these most basic elements [20], [21]. In the extremely complicated mammalian brains, it is so far only possible to analyze such networks for extremely limited regions that do not give a picture of how the system behaves as a whole [22]. The relative simplicity of the nervous system of *C. elegans* allows a detailed analysis of the network, defined in terms of both electrical (gap junctional) and chemical (synaptic) connections between the neurons (Fig. 1).

The ubiquity of modularity in brain networks leads to the obvious question about how to explain the evolution of such a structural organization [23]. One possible reason for the existence of modular architecture is that it may result in low average path length (which is associated with high efficiency of signal communication) and high clustering (that allows local segregation of information processing) in networks [24]. An alternative possibility is that segregation of neurons into spatially localized communities minimizes the total cost associated with the wiring length (the physical distance spanned by connections between neurons). This cost arises from resources associated with factors such as wiring volume as well as metabolism required for maintenance and propagation of signals across long distances [25]. Developmental constraints, such as the lineage relations between different neurons may also play an important role in determining the connection topology of the neuronal network [26]. In addition, the existence of empirically determined circuits responsible for specific functions (such as, movement associated with exploratory behavior, egg laying, etc.) in the *C. elegans* nervous system, raises the intriguing possibility that structurally defined modules are associated with definite functional roles [27]. The invariant neuronal connectivity profile of *C. elegans* allows us to explore the contributions of the above mentioned structural, developmental and functional constraints in governing the mesoscopic organization of the nervous system.

In this paper, we begin our analysis of the organization of the *C. elegans* nervous system by identifying structurally defined modules in the network of neurons linked by synapses and gap-junctions. Next, we investigate whether the observed modular structure can be explained by using arguments based on universal principles. Such criteria, which include minimizing the cost associated with neuronal connections [25], [28] and their genetic encoding [29], or, decreasing the signal propagation path [30], [31], have recently been proposed to explain observed patterns of neuronal position and connectivity. Complementing the above studies, we seek to understand the factors determining the topological arrangement of the nervous system, given the physical locations of the neurons. We determine the role of physical proximity between a pair of neurons in deciding the connection structure, by investigating the correlation between their spatial positions and their modular membership. We also compare these modules with the existing classification of the nematode nervous system into several ganglia, as the latter have been differentiated in terms of anatomical localization of their constituent neurons. Results from the above analysis suggests that resource constraints such as wiring cost cannot be the sole deciding factor governing the observed meso-level organization. We also show that the modules cannot be only a result of the common lineage of their member nodes.

It is natural to expect that the structure of the nervous system is optimized to rapidly process signals from the environment so that the organism can take appropriate action for its survival [32]. By looking at the deviation between the actual network and a system optimized for maximal communication efficiency in conjunction with minimum wiring cost, we infer the existence of additional functional constraints possibly related to processing of information (i.e., other than rapid signal transmission). Information processing refers to the transformation of signals [33], such as selective suppression of activation in specific pathways, which allows different sensory stimuli to initiate response in distinct sets of motor neurons. Absence of such transformations would result in non-specific arousal of a large number of connected neurons, and will not achieve the high level of control and coordination necessary for performing a variety of specific functional tasks. This is also related to the observation of relatively high clustering in *C. elegans* neuronal network as compared to other information networks (e.g., electronic logic circuits [34]). Previous investigation of the role of clustering in the performance of neural network models, viz., in associative recall of stored patterns, has shown that lower clustering exhibits much better performance [35]. It has also been shown that the clustering in *C. elegans* neuronal network is higher than that in degree-conserved randomized networks having the same wiring cost [31]. Therefore, the presence of enhanced clustering in a system that has evolved under intense competition for survival may imply it plays a key role in processing information.

This brings us to the possibility that the observed distribution of neurons among modules is closely related to the behavioral requirements of the organism. For this purpose, we investigate the relation between the modules and the different functional circuits governing specific behavioral aspects of *C. elegans*. We identify the functional importance of certain key neurons by observing their relative connectivity within their module as compared to that with neurons belonging to other modules. By looking at the correlation between local and global connectivity profiles of individual neurons, we observe that the nematode nervous system is different from systems designed for rapid signal transfer, including other information networks occurring in the technological domain, such as the Internet. Further, in contrast to previous observations on the similarity between biological signalling networks having different origins [36], [37], we find that the *C. elegans* neuronal network has properties distinct from at least one other biological network involved in signalling, the protein interaction network (viz., in terms of the assortativity and the role-to-role connectivity profile) [38], [39].

Thus, the analysis of the network at the mesoscopic level provides an appropriate framework for identifying the roles that different classes of constraints (developmental, structural and functional) play in determining the organization of a nervous system. It allows us to infer the existence of criteria related to processing of information governing the observed modular architecture in *C. elegans* neuronal inter-connections. It also provides the means for identifying neurons having key roles in the behavioral performance of the organism exclusively from anatomical information about their structural connectivity. Our results can help experimentalists in focusing their attention to a select group of neurons which may play a vital part in as yet undetermined functions.

## Results

### Modular Structure of the *C.elegans* Somatic Nervous System

We begin our study of the mesoscopic organization of the network by focusing on identifying its modular arrangement. In order to determine the community structure of the *C. elegans* neuronal network, we perform an optimal partitioning of the system into modules, that corresponds to the maximum value of modularity parameter, 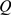 (see Methods for details on modularity measure 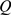 and the algorithm used for modularity determination). We have considered different cases corresponding to the different types of neuronal connections (viz., gap junctions and synapses) and the nature of such connections (i.e., unweighted or weighted by the number of connections between a given pair). While the gap junctional network is undirected, the directional nature of signal propagation through a synapse implies that the synaptic network is directed. For each network, we have obtained using the spectral method, the maximum modularity 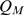 and the corresponding number of partitions (Table 1). Note that, the number of modules and their composition is dependent on the type of connections we consider.

**Table 1:** Modularity of the *C. elegans* neuronal network.

Network	Un-weighted						Weighted					
	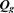	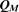	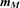	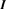	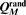	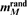	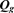	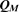	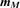	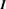	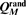	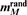
Gap Jn	0.207	0.630	11	0.326	0.467 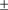 0.010	10.5 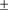 2.2	0.170	0.657	15	0.347	0.519 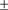 0.022	12.5 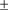 3.9
Synaptic	0.149	0.349	2	0.257	0.192 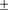 0.013	3.6 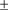 0.7	0.211	0.472	4	0.314	0.307 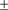 0.018	5.6 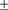 1.4
Combined	0.169	0.378	3	0.306	0.156 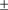 0.012	3.2 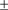 0.7	0.203	0.491	6	0.376	0.258 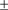 0.015	4.9 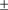 1.5

The modularity of the network is measured using the parameter 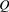, which requires a knowledge of the partitions or communities which divide the network. We obtain the modularity measure, 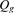, on assuming the communities to correspond to the ganglia. Its positive values indicate that neurons in the same ganglion have high density of inter-connections. We have also obtained 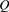 by determining the modules of the network using a spectral method, the corresponding values being indicated by 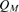. The relatively high values of 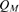 compared to 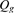, indicates that the ganglia do not match with this optimal partitioning of the network. The measures, 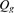 and 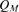, as well as the number of modules, 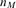, have been obtained for both unweighted and weighted networks consisting of either gap junctions or synapses or both. We calculate the overlap between the ganglionic and the optimal partition of the network using the normalized mutual information index, 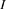. For the case of perfect match between the two, the index, 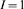, whereas if they are independent of each other, 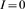. The measured values of 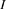 indicate that the overlap between the different modules and the anatomically defined ganglia is not high. The modular nature of the somatic nervous system is emphasised by comparing the empirical network with networks obtained by randomizing the connections, keeping the degree of each neuron fixed. The mean and standard deviation of the modularity 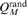 and the corresponding number of partitions 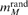 are shown for both weighted and unweighted networks, and for the different types of connections. For all cases, the randomized networks show a significantly lower modularity than the empirical network.

**Figure 2:**
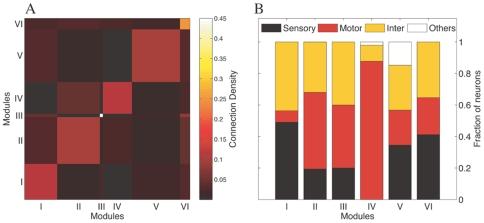
Modular interconnectivity and decomposition according to neuron type. (A) Matrix representing the average connection density between neurons occurring within modules and those in different modules. The figure indicates that neurons within a module are densely interconnected compared to the overall connectivity in the network. (B) The modules are decomposed according to the different neuron types comprising them. The figure shows that the modules are not simply composed of a single type of neuron.

As we want to consider all connections in our study, we have also worked with an aggregate network that includes synapses as well as gap junctions. Throughout this paper, we have reported results for this weighted combined network, unless stated otherwise (see Text S1 for the analysis of the network of synaptic connections). The link weights in the combined network correspond to the total number of synaptic and gap junctional connections from one neuron to another. The high value of 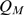 and dense inter-connectivity within modules (Fig. 2, A) suggest that the network has a modular organization (Table S1). We further validate our results by calculating the modularity of randomized versions of the network where the degree of each node is kept fixed (see Methods for the network randomization procedure). The average modularity of these randomized networks is considerably lower than that of the empirical network. We examine the robustness of the modular partitioning with respect to the method used for detecting communities by using another module determination algorithm [40]. However, this produces a lower value for 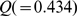 compared to the spectral method. The three modules identified using this alternative method have a substantial overlap (normalized mutual information 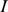 = 0.496) with the six modules obtained using the spectral method. It suggests that the alternative algorithm yields a coarser-grained picture of the network communities that are considered in the rest of the paper.

The modules do not have a simple relation with the anatomical layout of the worm. In particular, they are not a result of a simple division of the nervous system into groups responsible for receiving sensory input, and other groups involved in motor output. In Fig. 2 (B), we have analyzed the composition of the different modules in terms of distinct neuron types (viz. sensory, motor and inter-neurons). None of the modules are exclusively composed of a single type of neuron, although motor neurons do tend to dominate one module.

### Modules and Spatial Localization

To understand why modular structures occur in the neuronal network, we first consider the relation between the optimal partitions and the spatial localization of neurons in each module. This can tell us whether constraints related to the physical nearness between neurons, such as minimization of the wiring length, dictate the topological organization of the network. Wiring cost has already been shown to be the decisive factor governing *neuron positions* in the body of *C. elegans* [25], [28]. Thus, a plausible hypothesis is that, if most neuronal connections occur within a group of neurons which are physically adjacent to each other, then the wiring cost will be significantly decreased. In terms of connectivity, this will be manifested as a modular organization of the network, where each module will mostly comprise neurons in close physical proximity.

**Figure 3:**
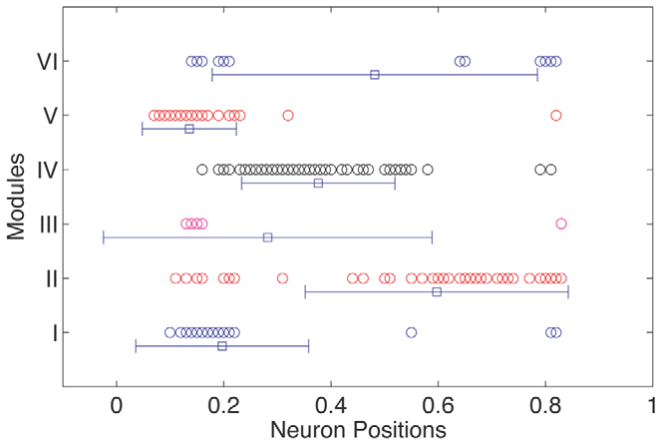
Neuronal layout of the worm indicating cell body positions of each neuron. The position of neuronal cell bodies along the longitudinal axis of the *C. elegans* body plan is shown, with the vertical offset and color indicating the module to which a neuron belongs. The mean and standard deviation of neuronal positions for each module is also indicated, suggesting relative absence of spatial localization in the modules.

Fig. 3 indicates the spatial location of the cell body for each neuron on the nematode body (along the longitudinal axis), segregated according to their membership in different modules. We see that a large fraction of the neurons belonging to the same module do indeed have their cell bodies close to each other. A one-way ANOVA test, comparing the positions of the neurons in the different modules with the null hypothesis that they are drawn from the same population, shows that it can be rejected at confidence level of 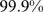. However, the large standard deviations for the distribution of positions of the module components reveal that none of the modules are spatially localized at any specific region on the nematode body axis. A more detailed multiple comparison procedure carried out for every pair of modules shows the absence of statistically significant spatial separation for the module pairs (I, III), (I, V), (II, VI), (III, IV), (III, V), (III, VI) and (IV, VI). The lack of significant distinction between the modules in terms of their physical location weighs against the hypothesis of wiring length minimization being the dominant factor governing the connectivity.

**Figure 4:**
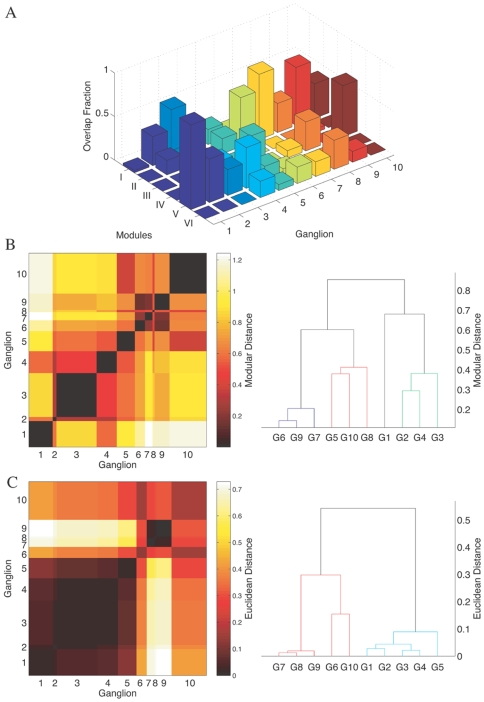
Modular decomposition of neurons in different ganglia. (A) Neurons belonging to different ganglia are decomposed according to their modular membership. The height of each bar in the histogram corresponds to the overlap between the ganglia and the modules, calculated as the fraction of neurons that are common to a particular ganglion and a specific module. (B) The matrix representing the average modular distance between the different ganglia, as calculated from the modular decomposition spectrum of each ganglion. The corresponding dendrogram indicates the closeness between different ganglia in the abstract 6-dimensional “modular” space. (C) The matrix of physical distances between the ganglia is shown for comparison with (B), calculated as the average distance between neurons belonging to the different ganglia. The corresponding dendrogram indicates the closeness between ganglia according to the geographical nearness of their constituent neurons in the nematode body. The difference indicates that the ganglia which are geographically close may not be neighbors in terms of their modular spectra.

The above conclusion is further supported by analysing the connectivity pattern of the different ganglia of the nematode nervous system. The nine anatomically defined ganglia (G1: Anterior, G2: Dorsal, G3: Lateral, G4: Ventral, G5: Retrovesicular, G6: Posterolateral, G7: Preanal, G8: Dorsorectal and G9: Lumbar), in addition to the ventral cord (G10), are defined in terms of physical proximity of their component neurons. Thus, a lower total connection length between neurons would result in the ganglia having a relatively higher density of connections between their constituent neurons. This would imply that the existence of ganglia imposes a modular structure in the connection topology. To examine how well the ganglionic arrangement explains the observed modularity of the neuronal network, we have measured the modularity value 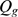 where the network communities correspond to the different ganglia. Although the non-zero value of 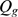 indicates that the connection density between the neurons in a ganglion is higher than that for the overall network, it is not as high as the maximum possible value of 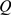 (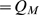, obtained for the optimal partitioning) as seen from Table 1. To measure the overlap between the modules obtained by optimal partitioning of the network and the ganglia, we calculate the normalized mutual information index, 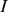 (see Methods). In the case of perfect match between the two, 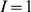, while, if there is no overlap, 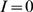. The low values for 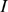 given in Table 1 suggest that the composition of the different ganglia is quite distinct from that of the modules. The overlap between the modules and the ganglia is shown explicitly in Fig. 4 (A), indicating that most ganglia are composed of neurons belonging to many different modules.

This distribution of the neurons of each ganglion into the 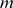 different modules of the optimal partition allows us to define a *modular decomposition* spectrum for the different ganglia. It gives us a metric for inter-ganglionic distance in a 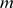-dimensional “modular” space. Thus, in this abstract space, two ganglia are close to each other if they have similar spectral profiles. Their distance in this “modular” space (Fig. 4, B) are then compared to their physical distance, as measured in terms of the average separation between the cell bodies of all pairs of neurons 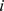 and 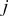, belonging to different ganglia (Fig. 4, C). The comparison of the two distance matrices shows that there are indeed certain similarities between these two different concepts of closeness between the ganglia. For example, the five ganglia located in the head (G1–G5) cluster together, as do the three located towards the tail (G7–G9). This similarity can be quantified by computing the correlation between these two distance measures (i.e., Euclidean and modular), 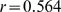 (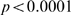). Our observation is in accord with previous reports which use the notion of wiring cost for explaining (to a certain extent) the observed relative positions of the ganglia [41]. However, when we consider the corresponding dendrograms that indicate the relative proximity of the different ganglia in physical space and in “modular” space, we observe significant differences between the two. Ganglia which are close to each other in physical space may not be neighbors in terms of their modular spectra. For instance, G5 which is located in the head, is closer in “modular” space to the ganglion G8 located in the tail. On computing the correlation coefficient between the two trees by considering the distances between every pair of ganglia (measured as the path length between the pair in the dendrogram), we obtain 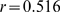 (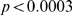). This value is substantially lower than 1, the value expected had the two dendrograms been identical. It reiterates our previous conclusion that wiring cost minimization, which is related to the physical distance between neurons, is not a dominant factor governing the organization of *C. elegans* somatic nervous system.

### Modules and Cell Lineage

As developmental processes are believed to play a critical role in determining the structure of the nervous system, we also consider the alternative hypothesis that the structural modules reflect a clustering of neurons that are related in terms of their lineage. Lineage of a cell is the pattern of successive cellular divisions that occur during its development. This is invariant in *C. elegans*, allowing one to trace the individual developmental history of each cell in order to identify the cell-autonomous mechanisms and inter-cellular interactions that define its fate [3]. We investigate whether a relation exists between the modular structure and the sequence of cell divisions that occur during development, by measuring the average relatedness between neurons occurring in the same module and comparing with that for neurons occurring in different modules.

**Figure 5:**
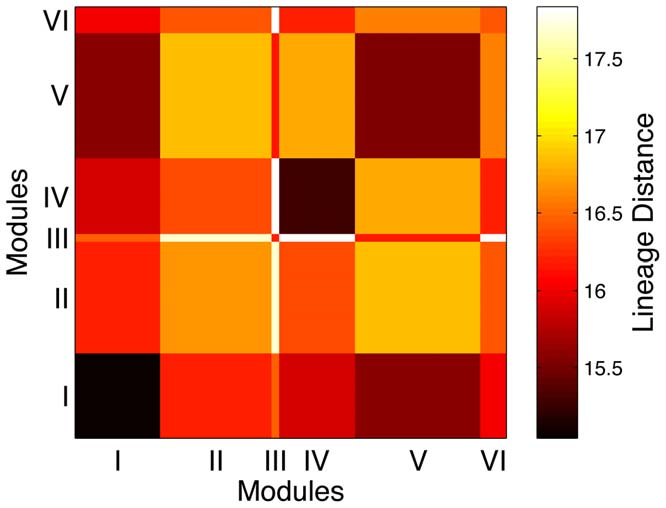
Lineage distance between modules. The matrix representing the average lineage distance between neurons occurring within the same module and those belonging to different modules. The figure indicates that neurons occurring in the same module have only a slightly lower lineage distance as compared to that between neurons occurring in different modules.

Fig. 5 indicates that it is difficult to distinguish the modules in terms of the lineage of the neurons comprising them. Indeed, even coarse distinctions such as AB and non-AB lineage neurons are not apparent from the modular division. A one-way ANOVA test, with the null hypothesis that the average lineage distance between neurons within the same module and that between neurons belonging to different modules are obtained from the same distribution, shows that it cannot be rejected at confidence level of 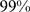. We next analyse each pair of modules using a multiple comparison procedure for the intra-modular and inter-modular lineage distances of their constituent neurons. This reveals that the neurons belonging to modules III and VI have within-module lineage distance distribution that is statistically indistinguishable from the distribution of their lineage distance with neurons belonging to any of the other modules. Thus, for at least two of the modules, one cannot segregate them in terms of cell lineage. The detailed view of the relatedness between each pair of neurons shown in Fig. S1 indicates that, while in each module there are subgroups of closely related neurons, different subgroups within the same module may be very far from each other in the lineage tree. Conversely, neurons occurring in different modules can have small distance between each other in terms of lineage. This observation is supported by the low correlation 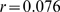 (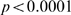) between the physical and lineage distances of neurons. The fact that *C. elegans* neurons are largely non-clonally derived from many different parent cells [42] may partly explain this lack of correlation between lineage and modules. The above results indicate that developmental constraints arising from common ancestry are not exclusively responsible for the observed connection structure of the *C. elegans* neuronal network.

### Optimizing between Wiring Cost and Communication Efficiency

In the previous sub-sections, we have shown that neither wiring cost minimization nor lineage considerations can by themselves determine the connection topology of the network. In order to ascertain the possible nature of the additional constraints that gives rise to the observed mesoscopic structure, we now investigate global properties of the neuronal network. A possible governing factor for network organization is that, rather than decreasing the total wiring length or the average physical distance between connected neurons, the network minimizes the path length for information transfer. This can be measured by the number of links that must be traversed to go from one neuron to another using the shortest route [30]. We consider this possibility by measuring the communication efficiency of the network, using the harmonic average path length between all pairs of neurons (see Methods).

**Figure 6:**
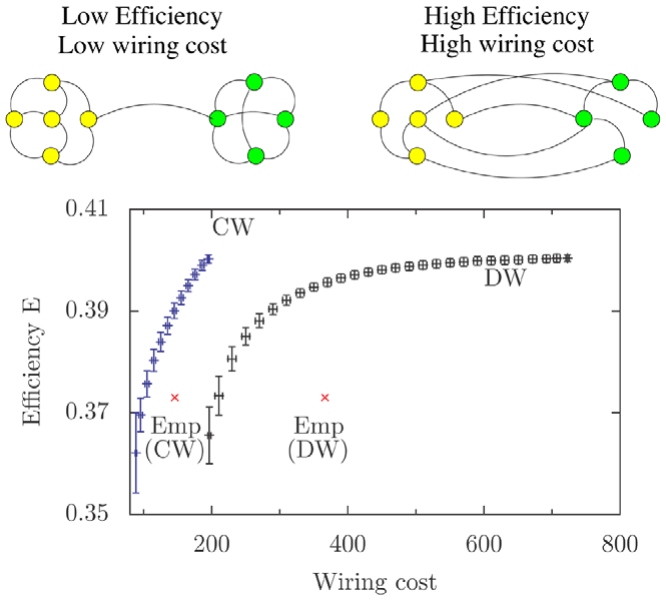
Trade-off between wiring cost and communication efficiency in the network. The variation of communication efficiency, 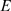, as a function of the wiring cost, defined using either the “dedicated-wire” model (DW) or the “common-wire” model (CW), in the ensemble of random networks with degree sequence identical to the *C. elegans* neuronal network. The trend indicates a trade-off between increasing communication efficiency and decreasing wiring cost. The corresponding values for the empirical network are indicated by crosses for both DW and CW. The schematic figures shown above the main panel indicate the type of networks obtained in the limiting cases when only one of the two constraints are satisfied. In both curves, error bars indicate the standard deviations calculated for 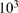 random realizations. We observe that the empirical network is suboptimal in terms of wiring cost and communication efficiency, suggesting the presence of other constraints governing the network organization.

It is evident that increasing the efficiency requires topological long-range connections, which however increases the wiring cost of the network (Fig. 6). Therefore, it is natural to expect that the system would try to optimize between these two constraints. Thus, we compare the performance of the network as a rapid signal propagation system against the resource cost for the required number of connections. This cost is measured as the Euclidean length between the cell bodies of all connected pairs of neurons, corresponding to the “dedicated-wire” model of Ref. [25]. It has been shown that the positions of the subset of sensory and motor neurons directly connected to sensory organs and muscles, respectively, can be determined quite accurately by minimizing their total wiring cost [28]. As our focus is on the connection structure of the neuronal network, we keep the neuron positions invariant. By randomizing the network, keeping the degree of each node unchanged, we can construct a system with a specific wiring cost. We then measure its communication efficiency. Fig. 6 reproduces the expected result that, decreasing the wiring cost of the network causes a decline in its performance in terms of its ability to propagate signals rapidly. However, it is surprising that the empirical network has a wiring cost much higher than that of the corresponding randomized network having the same communication efficiency. To see whether this could be an artifact of the measure used to calculate wiring cost, we have considered an alternative method for quantifying it.

Most of the neurons in *C. elegans* have at most one or two extended processes, on which all the synapses and gap junctions with other neurons are made. Thus the “dedicated-wire” definition of wiring cost that sums Euclidean distances between every connected neuronal pair may be a gross over-estimate of the actual usage of resources used in wiring. Instead, we can use a “common-wire” model to define the wiring cost for connecting to a specific neuron. This is measured by the Euclidean distance between the neuron's cell body and that of the farthest neuron (along the longitudinal axis) it is connected to. The simple one-dimensional simplification of the *C. elegans* body that we have assumed here ignores distance along the transverse plane. Thus, this measure is actually an under-estimate of the actual wiring cost, and should provide an insightful comparison with the above measure obtained from the “dedicated-wire” model. Fig. 6 (inset) shows that wiring cost increases with communication efficiency for the randomized networks, which is qualitatively similar to the relation obtained using the preceding definition for wiring cost. In this case also, we find that the empirical *C. elegans* network has a much lower efficiency in comparison with an equivalent randomized network having the same wiring cost. Thus, as this observation is independent of these two definitions of wiring cost, it suggests the presence of other constraints that force the neuronal network to have a higher wiring cost or lower efficiency than we would have expected. These constraints are possibly related to information processing, which is the principal function of the nervous system.

### Information Processing Is a Distinctive Feature of the *C. elegans* Neuronal Network

To explore further the possibility that the additional constraints governing the topological structure of *C. elegans* nervous system may be related to information processing, we investigate how this functional requirement could be responsible for differentiating the system from other complex networks for which communication efficiency is of paramount importance. Rapid communication of information between different neurons is certainly an important performance criterion. However, the neuronal network has properties quite distinct from that of (say) the Internet or the airline transportation network, which are systems designed for maximum transportation efficiency of signals or physical resources.

For this purpose, we look at the overall network design by decomposing the system into (i) a strongly connected component (SCC), within which it is possible to visit any node from any other node using directed links, (ii) an inward component (IN) and (iii) an outward component (OUT), consisting of nodes from which the SCC can be visited or which can be visited from the SCC, respectively, but not vice versa. In addition, there can be disconnected components, i.e., nodes which cannot be visited from SCC nor can any visits be made to SCC from there (Fig. 1). A comparison of the *C. elegans* neuronal network with a similar decomposition of the WWW [43] reveals that, while in the latter the different components are approximately of equal size (WWW: SCC
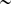
56 million pages, while IN, OUT consist of 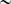
43 million pages each), the SCC of the nervous system comprises almost the entire network (SCC has 274 neurons, IN has 4 neurons and OUT has 1 neuron). Thus, any node can, in principle, affect any other node in the nervous system, suggesting the importance of feedback control for information processing.

**Figure 7:**
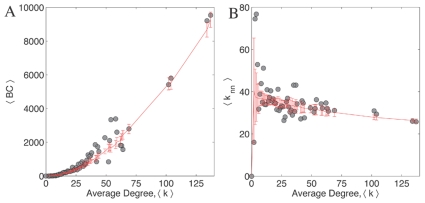
Betweenness centrality and the average nearest neighbor degree as a function of the total degree of network. (A) The average betweenness centrality, 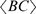, and (B) the average nearest neighbor degree, 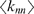 of each node as a function of its total degree, 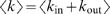. Betweenness centrality is a measure of how frequently a particular node is used when a signal is being sent between any pair of nodes in the network using the shortest path. In case of the Internet, BC of nodes increases with its degree which is sought to be linked with its information transport property. In *C. elegans*, although BC increases with degree, this increase is not significant when compared to the randomized version of the network. In the case of the relation between the average connectivity of nearest neighbors of a node with its total degree 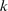, we note that for both the Internet and protein interaction network, 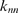 decreases with 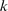 as a power law. This means that low connectivity nodes have high degree nodes as their neighbors and vice-versa. However, in the case of *C. elegans*, this relation is not very apparent and insignificant in comparison with the randomized version of the network. In both figures, error bars indicate the standard deviations calculated for 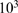 random realizations. These results suggest that the *C. elegans* network forms a class distinct from the class of networks optimized only for signal propagation.

Next, we consider the relation between two fundamental properties of the network: the degree of nodes and their *betweenness centrality* (BC), which characterizes the importance of a node in information propagation over the network (see Methods). We observe that for both the *C. elegans* neuronal network and its randomized versions, the degree of a node and its BC are strongly correlated, i.e., highly connected nodes are also the most central (Fig. 7, A). This is similar to what has been observed in the Internet [44], where the highest degree nodes are also those with the highest betweenness [45], but in sharp contrast to the airport transportation network, where non-hub nodes (low degree) may have very large BC [46].

However, the *C. elegans* neuronal network differs from other networks whose primary function is to allow signal propagation between nodes (viz., the Internet and the protein interaction network (PIN)), in terms of the variation of the degree of a node with the average degree of its neighboring nodes, 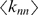. While in the Internet and PIN, 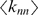 decays as a power law with node degree, in the neuronal network, this dependence is very weak (Fig 7, B), especially when contrasted with the randomized ensemble. This implies that the *C. elegans* nervous system does not have multiple star-like subnetworks as seen in the Internet and PIN [39]. Further, it is different from the airline transportation network, where the high degree nodes are closely connected among themselves, showing an assortative behavior [47]. In fact, computation of the assortativity coefficient 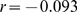 indicates that the network is disassortative (as previously stated in Refs. [48], [49]), although comparison with that of the degree-conserved randomized ensemble (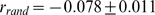) indicates that this is predominantly a result of the degree sequence. Another important distinguishing characteristic of the *C. elegans* network is that the distributions of link weight and degree do not appear to be scale-free, or even having a long tail (Fig. S2), unlike systems such as the Internet and the airline transportation network [47], [50]. Thus, our study shows that there are additional constraints governing the nervous system connection topology in *C. elegans*, which are unrelated to wiring cost, lineage or communication efficiency. As the principal function of the system is to process information, the above results suggest it is this functional requirement that provides the additional constraints leading to the observed organization of the nematode neuronal network.

### Modules and Functional Circuits

In order to understand the nature of functional considerations that may govern the network organization, we focus on the overlap of several previously identified functional circuits of *C. elegans* with the structurally identified modules. Functional circuits are a subset of neurons which are believed to play a vital role in performing a function, and are distinguished by observing abnormal behavior of the organism when they are individually removed (e.g., by laser ablation). In biological systems, it has been observed that members of structurally defined communities are often functionally related (e.g., in the intra-cellular protein interaction network [13] and the network of cortical areas in the brain [51], [52]). Here, we investigate the possibility of a similar correlation between the anatomical modules of *C. elegans* and its functional circuits. We consider the functional circuits for (F1) mechanosensation [53]–[55], (F2) egg laying [56], [57], (F3) thermotaxis [58], (F4) chemosensation [59], (F5) feeding [2], [53], [60], (F6) exploration [2], [53], [60] and (F7) tap withdrawal [54], [61] (Table S2).

**Figure 8:**
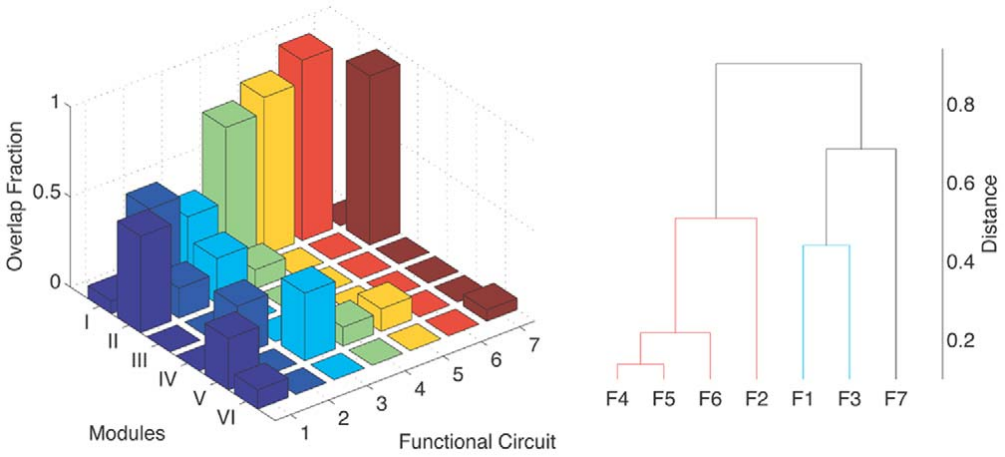
Modular decomposition of neurons in different functional circuits. Neurons belonging to different functional circuits are decomposed according to their modular membership. The height of each bar in the histogram corresponds to the overlap between the modules and functional circuits (F1) mechanosensation, (F2) egg laying, (F3) thermotaxis, (F4) chemosensation, (F5) feeding, (F6) exploration and (F7) tap withdrawal. The overlap is measured in terms of the fraction of neurons common to a particular functional circuit and a specific module. The corresponding dendrogram represents the closeness between different functional circuits in the abstract 6-dimensional “modular” space.

Fig. 8 shows the modular decomposition of each functional circuit, indicating their overlap with the modules. The corresponding dendrogram clusters the circuits in terms of the similarity in their modular spectra. We note that the circuits for chemosensation, feeding and exploration are clustered together. This is consistent with the fact that most of the neurons belonging to the feeding and exploration circuits are involved in chemosensation. A surprising observation is that although F6 is a subset of F4, it is actually closer to F5 in “modular” space (distance = 0.18) than to F4 (distance = 0.26), despite the feeding and exploration circuits not having any neuron in common. This close relation between F5 and F6 in modular space is suggestive of a relation between modularity and functionality, as it is known from experiments that there is a strong connection between their corresponding functions. The feeding behavior of *C. elegans* is known to be regulated in a context-dependent manner by its chemical milieu. It integrates external signals [62], such as the availability of food, and nutritional status of the animal, to direct an appropriate response [63]. An example is the avoidance of high CO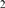 concentrations by satiated animals [64]. Further, the mode of locomotion of the organism is also determined by the quality of food [65]. Another important observation made from the modular decomposition is the proximity of the functional circuit F2 to the group (F4,F5,F6). This is significant in light of experimental observations that presence of food detected through chemosensory neurons modulates the egg-laying rate in *C. elegans* [55], [66]. The above results indicate that the relation between the functional circuits, which are essential for the survival of the organism, are reflected in the modular organization of the nematode nervous system.

### Functional Roles of Different Neurons

Having looked at the functional circuits and the relations between them in the previous section, we now investigate the importance of individual neurons in terms of their connectivity. This is revealed by a comparison between the localization of their connections within their own community and their global connectivity profile over the entire network. In order to do this, we focus on (i) the degree of a node within its module, 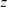, that indicates the number of connections a node has to other members of its module, and (ii) its participation coefficient, 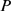, which measures how dispersed the connections of a node are among the different modules [39].

A node having low within-module degree is called a non-hub (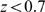) which can be further classified according to their fraction of connections with other modules. Following Ref. [39], these are classified as (R1) ultra-peripheral nodes (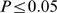), having connections only within their module, (R2) peripheral nodes (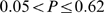), which have a majority of their links within their module, (R3) satellite connectors (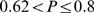), with many links connecting nodes outside their modules, and (R4) kinless nodes (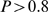), which form links uniformly across the network. Hubs, i.e., nodes having relatively large number of connections within their module (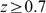), are also divided according to their participation coefficient into (R5) provincial hubs (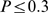), with most connections within their module, (R6) connector hubs (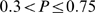), with a significant fraction of links distributed among many modules, and (R7) global hubs (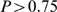), which connect homogeneously to all modules. This classification allows us to distinguish nodes according to their different roles as brought out by their intra-modular and inter-modular connectivity patterns (Table S3).

**Figure 9:**
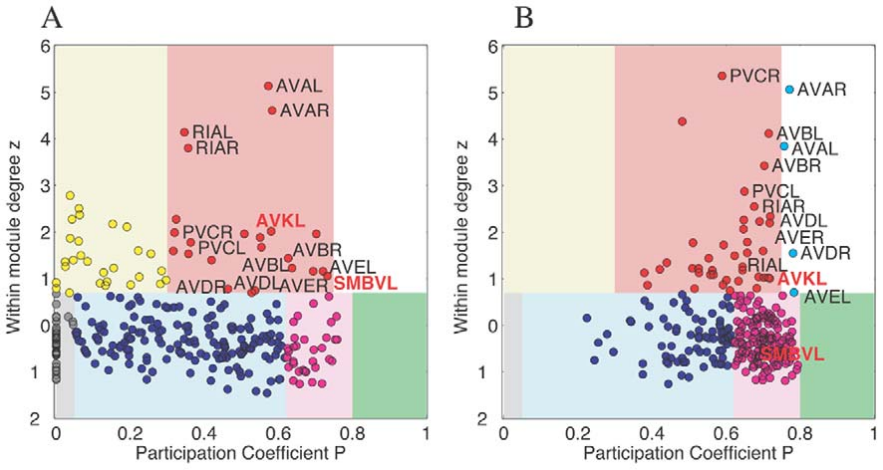
The role of individual neurons according to their intra- and inter-modular connectivity. (A) The within module degree 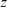-score of each neuron in the empirical neuronal network is shown against the corresponding participation coefficient 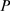. The within module degree measures the connectivity of a node to other nodes within its own module, while the participation coefficient measures its connectivity with neurons in the entire network. (B) The corresponding result for a randomized version of the *C. elegans* network where the degree of each neuron is kept unchanged is also shown. Neurons belonging to the different regions in the 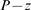 space are categorised as: (gray) R1: “ultraperipheral nodes”, i.e., nodes with all their links within their module, (blue) R2: “peripheral nodes”, i.e., nodes with most links within their module, (pink) R3: “nonhub connector nodes”, i.e., nodes with many links to other modules, (green) R4: “nonhub kinless nodes”, i.e., nodes with links homogeneously distributed among all modules, (yellow) R5: “provincial hubs”, i.e., hub nodes with the vast majority of links within their module, (red) R6: “connector hubs”, i.e., hubs with many links to most of the other modules, and (white) R7: “global hubs”, i.e., hubs with links homogeneously distributed among all modules. The neurons occurring as connector hubs are identified in the figure. Most of these neurons occur in different functional circuits indicating the close relation between functional importance and connectivity pattern of individual neurons. In addition, the neurons AVKL and SMBVL which are predicted to be functionally important are separately marked.

We will now use the above methodology on the *C. elegans* network in order to identify neurons that play a vital role in coordinating activity through sharing information (either locally within their community or globally over the entire network). Fig. 9 shows the comparison between the empirical network and a corresponding randomized network (obtained by keeping the degree of each node fixed). Results for a randomized ensemble, comparing the number of neurons in each role against that for the empirical network, are given in Table S4. We immediately notice that the randomized networks have relatively very few nodes having the roles R1 and R5, indicating that the modular nature of the original network has been lost. In fact, in the randomized system, most nodes have higher participation coefficient, with a large majority being satellite connectors (R3). More interesting is the fact that, the empirical neural network does not possess any neuron having the global roles played by R4 and R7, whereas these regions may be populated in randomized networks. This implies that modular identity in the *C. elegans* neuronal network is very pronounced.

It is possible that, neurons having the role of provincial hubs may be involved in local coordination of neural activity, while, the connector hubs may be responsible for integration of local excitations to produce a coherent response of the entire system. This hypothesis is supported by noting that all command interneurons (of the class AVA, AVB, AVD, AVE, PVC), which control forward and backward locomotion of the worm by regulating motor output, play the role of connector hubs. In fact, out of the 23 neurons in the class R6, 20 are known to belong to different functional circuits. Among the rest, although DVA does not belong to any of the known circuits, it has been identified as being involved in mechanosensory response and in its absence, the frequency and magnitude of the tap-induced reversal, as well as the acceleration magnitude, is diminished [61]. The two remaining neurons, AVKL and SMBVL, have not been implicated so far in any known functional circuit. However, their occurrence in this class suggests that they may be important for some, as yet unknown, function. This is a potentially interesting prediction that may be verified in the laboratory.

The significance of these results is underlined by a comparison with the randomized network. For instance, in the random realization shown in Fig. 9 (B), of the 49 neurons playing the role of connector or global hubs, less than half (viz., 23) actually belong to any of the known functional circuits. The appearance of most of the command interneurons in the high-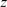 region of both the empirical and randomized networks indicates that their high overall degree is responsible for their observed role of “connecting hubs”.

We now turn to the 28 neurons which play the role of provincial hubs. Half of all the inhibitory D-class motorneurons (viz., DD1-DD3 and VD1-VD6) are found to belong to this class. This is significant as these neurons have already been implicated in the ability of the worm to initiate backward motion. While they also contribute to forward locomotion, previous experiments have shown that they are not essential [67]. This fits with our hypothesis that, R5 neurons are important for local coordination but may not be crucial for the global integration of activity. A pair of excitatory B-class motorneurons that sustain coordinated forward locomotion in the worm also appear as provincial hubs. Of the remaining R5 neurons, 9 have been previously identified as belonging to various functional circuits. It will be interesting to verify the functional relevance of the remaining 8 neurons (OLLL/R, RMDVL/R, SMDVR, RIH, RMDDL/R) in the laboratory. Thus, overall, we find a very good correlation between the connectivity pattern and the functional importance of different neurons.

Analysis of neurons having different roles in terms of their membership in the different ganglia (Fig. S3) indicates that the lateral ganglion provides the major fraction of neurons acting as connector hubs (R6). This is consistent with an earlier study where this ganglion was found to be the principal highway for information flowing between neurons responsible for receiving sensory stimuli and those involved in motor response [68]. To check the significance of the above result, we observe the membership of the set of connector hubs in the corresponding randomized network (Fig. S3). While this also shows many neurons from the lateral ganglion, unlike in the empirical network it has representation from other ganglia too (e.g., the retrovesicular ganglion).

We have also carried out an analysis of the frequency of links between neurons having different roles, relative to the randomized network (Fig. S4). This allows us to compare the *C. elegans* neuronal network with other networks involved in (a) transportation and (b) information propagation. It has been shown that networks of class (b) shows significant under-representation of links between R1-R1, R5-R6 and R6-R6, whereas networks of class (a) exhibit over-representation of all three [39]. These patterns have been related to the occurrence of stringy periphery in class (a) and multi-star structures in class (b) [39]. In the case of *C. elegans*, R1-R1 does seem to be over-represented. However, R6-R6 shows very little over-representation, while both R5-R6 and R6-R5 show slight under-representation. This difference in the role-to-role connectivity pattern for the nematode nervous system with the networks in the above-mentioned two classes suggests that its structure is not exclusively characterized by either a stringy periphery or multiple stars. This assumes significance in light of recent work distinguishing information (or signalling) networks, such as the Internet and protein interactome, on the one hand, and transportation networks, such as metabolic and airport networks, on the other, into two classes [39]. Our results suggest that neuronal networks which have to *process* information, in addition to transferring signals, may constitute a different category from either of the above classes.

## Discussion

In this paper, we have carried out a detailed analysis of the mesoscopic structure in the connection topology of the *C. elegans* neuronal network. Inferring the organizing principles underlying the network may give us an understanding of the way in which an organism makes sense of the external world. We have focused primarily on the existence of modules, i.e., groups of neurons having higher connection density among themselves than with neurons in other groups. The presence of such mesoscopic organization naturally prompts us to ask the reasons behind the evolution of these features in the network.

In lower invertebrates like nematodes, the genome is the dominant factor which governs the development of the organism, including its nervous system. The neuronal network structure is formed early in the life-cycle of the organism, when most of the cells and their connections are configured permanently. Although external cues may play a role, the relative absence of individual variations in the network organization makes *C. elegans* an ideal system for studying how the system has evolved to optimize for various constraints, such as minimizing resource use and maximizing performance.

There have been recent attempts at explaining neuronal position and structural layout of the network by using static constraints, such as wiring economy and communication path minimization. Although we find that membership of neurons in specific modules are correlated with their physical nearness, the empirical network is sub-optimal in terms of both the above-mentioned constraints. By comparing the system with other complex networks that have been either designed or have evolved for rapid transportation while being subject to wiring economy, we find that the *C. elegans* nervous system stands apart as a distinct class. This suggests that the principal function of neuronal networks, viz., the processing of information, distinguishes it from the other networks considered, and plays a vital role in governing its arrangement. Considering the importance of this constraint in ensuring the survival of an organism, it is natural that this should be key to the organizing principles underlying the design of the network. The intimate relation between function and structure of the nervous system is further brought out by our use of structural analysis to distinguish neurons that are critical for the survival of the organism. In addition to identifying neurons that have been already empirically implicated in different functions (which serve as a verification of our method), we also predict several neurons which can be potentially crucial for certain, as yet unidentified, functions.

Biological systems are distinguished by the occurrence of discrete entities with a distinct function, which are often termed as *functional* modules [9]. For several networks that occur in the biological context (such as that of protein interactions), the components of *structural* modules are seen to be functionally related. This suggests that modules provide a framework for relating mesoscopic patterns in the connection topology to subsystems responsible for specific functions. Although there is no unique correspondence between the structural modules of *C. elegans* and the known functional circuits [69], we use the overlap of the circuits with the modular membership of their constituent neurons to discover correlations between them. Our results reveal non-trivial association between circuits whose corresponding functions are closely connected, even when they do not share any common neurons. As such relations could not have been revealed by a micro-scale study which focuses on individual neurons and their connections, this result highlights one of the significant advantages of investigating the network at the mesoscopic level.

When we compare the nervous system of *C. elegans* with the brains of higher organisms, we observe the modular organization of the latter to be more prominent [70]. For example, the network of cortical areas in the cat and macaque brains exhibit distinct modules [24], [71], with each module being identified with specific functions [51], [52]. A possible reason for the relatively weak modular structure in the nematode could be due to the existence of extended processes for the neurons of *C. elegans*. Many of these span almost the entire body length, an effect that is enhanced by the approximately linear nature of the nematode body plan. As a result, connections are not constrained by the physical distance between soma of the neurons, as is mostly the case in mammalian brains. It is apparent that such constraints on the geographical distance spanned by links between nodes (viz., cost of wiring length) can give rise to clustering of connections among physically adjacent elements. In addition, the small nervous system of *C. elegans*, comprising only 302 neurons, lacks redundancy. Therefore, individual neurons may often have to perform a set of tasks which in higher organisms are performed by several different neurons. Thus, functional modularity is less prominent in the nematode as some neurons belong to multiple behavioral circuits.

Another principal distinction between the *C. elegans* nervous system and the brains of higher organisms such as human beings, is the relative high connectivity in the former (the connection density being 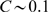). By contrast, the connectance for human brain is around 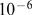 [72], [73], which leads us to the question of how communication efficiency can remain high in such a sparsely connected network. It is possible that the more intricate hierarchical and modular structures seen in the brains of higher organisms is a response to the above problem. The fact that the rate at which the number of neurons 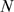 increase across species, is not matched by a corresponding increase in the number of links (which increases slower than 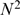 [74]) implies the existence of constraints on the latter, which is a resource cost in addition to the earlier mentioned cost of wiring length. Note that, in the present work we have focused on a single level of modular decomposition of the nematode neuronal network. It is possible that the system may have multiple levels of hierarchically arranged inter-nested modules [15], [75]. Investigating the existence of such organization in the *C. elegans* nervous system is a potential topic for future exploration.

Networks provide the scaffolding for the computational architectures that mediate cognitive functions. The pattern of connections of a neuron (or a neuronal cluster) defines its functionality not just locally but also as an integrated part of the nervous system. This is because neurocognitive networks across the evolutionary tree consist of interconnected clusters of neurons that are simultaneously activated for generating a single or a series of cognitive outputs. However, while some of the neurons (or clusters of neurons) are essential for the relevant outcome, others are ancillary. Thus, they work in a collaborative mode but are not interchangeable, each displaying relative specializations for separate behavioral components. Since the most prominent neuronal pathways are those that interconnect components of functional circuits, our analysis validates the intrinsic connection between network structure and the functions of the nervous system. The concept that behavioral consequences of damaging a region of the brain will reflect the disruption of the underlying network architecture may be intuitive but is particularly important when one considers brain dysfunction through neurodegeneration [76], where atrophy is seen to propagate preferentially through networks of functionally related neurons [77]. While the idea that damage in one part of the brain can affect other areas connected to that region is not new, our work on the mesoscopic network organization may be extended to look at disease progression through the network, beyond the current focus on the pathology within individual cells.

## Materials and Methods

### Connectivity Data

We have used information about the network connectivity, positions of neurons and the lineage distance between cells for the *C. elegans* nervous system, from the database published in Ref. [25] and available from the online Atlas of *C. elegans* anatomy (www.wormatlas.org/neuronalwiring.html#NeuronalconnectivityII).

This is an updated and revised version of the wiring data originally published in Ref. [2]. The connectivity between neurons and the positions of the neuron cell bodies along the longitudinal axis of the worm is reconstructed based on serial section electron micrography. Note that, the new database adds or updates about 3000 connections to the previous version. The lineage data indicates the relatedness between every pair of neurons in terms of distances in the embryonic and post-embryonic lineage trees. This is measured by identifying the last common progenitor cell of the two neurons, and then counting the number of cell divisions from this common ancestor. Each cell division adds a single unit to the total lineage distance, with the initial division from the common progenitor counted only once.

### Modularity (Q)

To decompose a given network into modules (where a module or community is defined as a subnetwork having a higher density of connections relative to the entire network) we use a method introduced in Ref. [78]. We compute a quantitative measure of modularity, 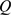, for a given partitioning of the network into several communities,
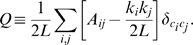
(1)Here, 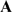 is the adjacency matrix (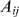 is 1, if neurons 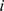, 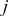 are connected, and 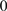, otherwise). The degree of each node 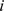 is given by 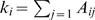. 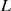 is the total number of links in the network, 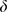 is the Kronecker delta (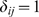, if 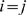, and 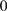, otherwise), and 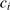 is the label of the community to which vertex 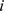 is assigned. In the case of directed and weighted network, the above measure can be generalized as
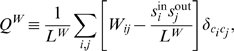
(2)where, 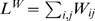 is the sum of weights of all links in the network (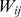 is the weight of the link from neuron 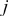 to neuron 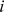), and the weighted in-degree and out-degree of node 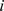 are given by 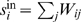 and 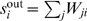, respectively.

The optimal partitioning of the network is the one which maximizes the modularity measure 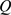 (or 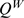). We obtain this using a generalization of the spectral method [79], [80]. We first define a modularity matrix 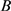,
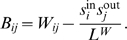
(3)To split the network into modules, the eigenvectors corresponding to the largest positive eigenvalue of the symmetric matrix (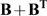) is calculated and the communities are assigned based on the sign of the elements of the eigenvector. This divides the network into two parts, which is refined further by exchanging the module membership of each node in turn if it results in an increase in the modularity. The process is then repeated by splitting each of the two divisions into further subdivisions. This recursive bisection of the network is carried out until no further increase of 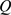 is possible.

### Modular Spectra

We analyze different neuronal groups, defined in terms of functions, anatomy (e.g., ganglia), etc., by proposing a decomposition in terms of the overlap of their constituent neurons with the different modules. Let the set of all neurons be optimally partitioned into 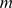 modules. We then define an overlap matrix, 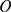, where the rows correspond to the different neuronal groups, and the columns correspond to the different modules. An element of this overlap matrix, 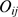, is the number of neurons in group 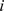 that are from the module 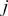. Then, the decomposition of the 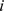-th group in the abstract 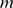-dimensional basis space formed by the modules is 
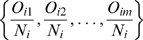
, where 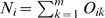 is the total number of neurons in the 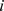-th group. The distance between two groups 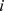 and 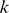 in this “modular” space is defined as
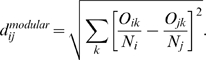
(4)Thus, this measure can be used as a metric for closeness or proximity between different neuronal groups.

### Decomposition of the Network into SCC, IN and OUT Components

In order to determine the Strongly Connected Component (SCC) of the network, we first calculate the graph distance matrix containing the shortest directed path between every pair of nodes in the network. A finite path length from neuron 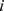 to neuron 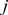 indicates the existence of a connected path from one neuron to the other. By grouping together all nodes which have finite path length with all other members of the group, we determine the SCC. In general, one can use Tarjan's algorithm for SCC determination [81]. Next, we identify all neurons not belonging to SCC but which can be reached via a directed path starting from a node in SCC. This constitutes the OUT component of the network. Similarly, the group of neurons which do not belong to SCC but which have finite directed path length to a member of SCC, constitutes the set of IN neurons.

### Betweenness Centrality (BC)

To measure the importance of a node in facilitating communication across a network, we consider how frequently a node is used to convey information from any part of the network to any other part using the shortest available path. The betweenness centrality of a node 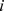 is defined as the fraction of shortest paths between the pairs of all other nodes in the network that pass through 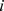 [82]. If the total number of shortest paths between nodes 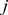 and 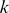 is 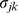, of which 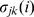 paths pass through node 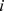, then the betweenness centrality of node 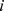 is
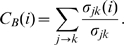
(5)


### Communication Efficiency (E)

To measure the speed of information transfer over the network, one can define the efficiency 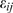 of communication between vertices 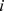 and 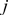 to be inversely proportional to the shortest graph distance 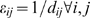. Therefore, the efficiency of communication across the whole network is
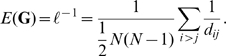
(6)This is the harmonic mean of graph distances between all pairs, which does not diverge even when the network is disconnected [83].

### Network Randomization

An ensemble of randomized versions of the empirical network is constructed keeping the in-degree and out-degree of each node unchanged. Each such network is created by rewiring randomly selected pairs of directed edges, 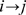 and 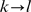, such that, in the randomized network, the corresponding directed edges are 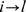 and 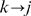. However, if these new links already exist in the empirical network, this step is aborted and a new pair of edges are chosen in order to prevent the occurrence of multiple edges [38]. The above procedure is repeated 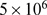 times for a single realization of the randomized network. In order to compare the properties of the empirical network with its randomized version, an ensemble of 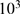 realizations is considered.

### Determining the Intra- and Inter-Modular Role of a Neuron

The role played by each neuron in terms of its connectivity within its own module and in the entire network is determined according to two properties [46]: (i) the relative within module degree, 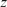, and (ii) the participation coefficient, 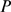.

The 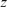-score of the within module degree distinguishes nodes that are hubs of their communities from those that are non-hubs. It is defined as
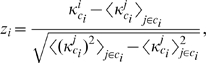
(7)where 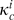 is the number of links of node 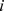 to other nodes in its community 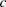 and 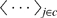 are taken over all nodes in module 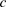. The within-community degree 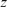-score measures how well-connected node 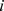 is to other nodes in the community.

The nodes are also distinguished based on their connectivity profile over the entire network, in particular, their connections to nodes in other communities. Two nodes with same within module degree 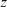-score can play different roles, if one of them has significantly higher inter-modular connections compared to the other. This is measured by the participation coefficient 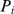 of node 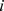, defined as
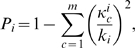
(8)where 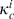 is the number of links from node 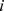 to other nodes in its community 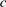 and 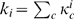 is the total degree of node 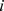. Therefore, the participation coefficient of a node is close to 1, if its links are uniformly distributed among all the communities, and is 0, if all its links are within its own community.

### Normalized Mutual Information (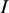)

To measure the overlap of the membership of nodes in a ganglion with their membership in a specific module, we use the normalized mutual information measure [84], [85]. First, we define a overlap matrix, 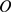, where the rows correspond to the different ganglia, and the columns correspond to the modules obtained by optimal partitioning of the network. An element of this overlap matrix, 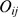, is the number of neurons in the ganglion 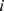 that appear in the module 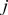. An information-theoretic measure of similarity between the partitions can then be defined as
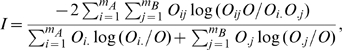
(9)where 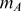 and 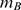 are the numbers of ganglia and modules respectively. The sum over row 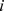 of matrix 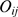 is denoted by 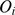 and the sum over column 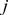 is denoted by 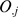. If the modules are identical to the ganglia, then 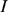 takes its maximum value of 1. On the other hand, if the modular partitioning is independent of the division of the network into ganglia, 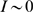.

## Supporting Information

Figure S1 Matrix representing the relatedness of neurons in the somatic nervous system of *C. elegans* as measured in terms of their lineage distance. The neurons are arranged according to the modules they belong to. The module boundaries are indicated in the figure. Within each module, neurons that are close in terms of lineage are placed in adjacent positions. The figure shows that closely related neurons may occur in different modules, while those in the same module may be far apart in terms of lineage distance. This indicates that there is no simple relation between relatedness of neurons in terms of lineage and their modular membership.
(0.05 MB PDF)

Figure S2 Cumulative distributions of the strength and (inset) degree for the (top) gap-junctional, (center) synaptic and (bottom) combined networks. The gap-junctional network is undirected and the strength of a node is defined as s_i_ = Σ_j_ W_ij_, where W_ij_ is the number of gap junctions between neurons i and j. On the other hand, the synaptic and combined networks are directed and the inward- and outward-strength of a node are defined as s_i_^in^ = Σ_j_ W_ji_, and s_i_^out^ = Σ_j_ W_ij_, respectively. For directed networks, W_ij_ represents the number of connections from neuron j to i. The figures indicate that scale-free behavior of the distributions is seen only for the gap-junctional network. The other two networks exhibit exponentially decaying nature for both the degree and the strength distributions.
(0.01 MB PDF)

Figure S3 The intra- and inter-modular connectivity of individual neurons in the *C. elegans* somatic nervous systems, color-coded to represent the different ganglia in which each occurs. (Top) The within module degree z-score of each neuron in the empirical neuronal network is shown against the corresponding participation coefficient P. (Bottom) The corresponding result for a randomized version of the *C. elegans* network where the degree of each neuron is kept unchanged. The lateral ganglion is seen to occupy a prominent position in the system, coordinating the information flow between the neuronal groups responsible for receiving sensory stimuli and those controlling motor activity. On the other hand, the randomized network shows similar prominence for several other ganglia.
(0.04 MB PDF)

Figure S4 The role-to-role connectivity pattern indicated by the z-scores for abundance of links between each pair of roles (R1–R7) in *C. elegans* neuronal network. Note that, as there are no neurons having roles R4 or R7 in the empirical network, links from other roles to these two do not exist. The z-scores represent the abundance of links between each pair of roles in the *C. elegans* somatic nervous system with respect to degree- and modularity-preserved randomized ensemble of networks (10^3^ realizations). The method used for calculating the z-score is as described in R. Guimera, M. Sales-Pardo and L.A.N. Amaral, “Classes of complex networks defined by role-to-role connectivity profiles”, Nature Physics, 3 (2007) 63–69.
(0.01 MB PDF)

Table S1 The classification of neurons according to their membership in the 6 modules obtained by optimal partitioning of the combined synaptic-gap junctional network of the *C. elegans* somatic nervous system.
(0.02 MB XLS)

Table S2 The neuronal composition of different functional circuits in the *C. elegans* somatic nervous system.
(0.02 MB XLS)

Table S3 The classification of neurons in the *C. elegans* somatic nervous system, according to their role in intra- and inter-modular connectivity.
(0.02 MB XLS)

Table S4 Comparison of the number of neurons in each role (R1–R7) between the empirical network and the degree-conserved randomized ensemble.
(0.01 MB XLS)

Text S1 Analysis of the *C. elegans* network of synaptic connections.
(0.16 MB PDF)
