# Supplementary figures and images for: Mesoscopic Organization Reveals the Constraints Governing Caenorhabditis elegans Nervous System
Source: PLoS One. 2010 Feb 22;5(2):e9240. doi: 10.1371/journal.pone.0009240 (PMC2825259; doi:10.1371/journal.pone.0009240)

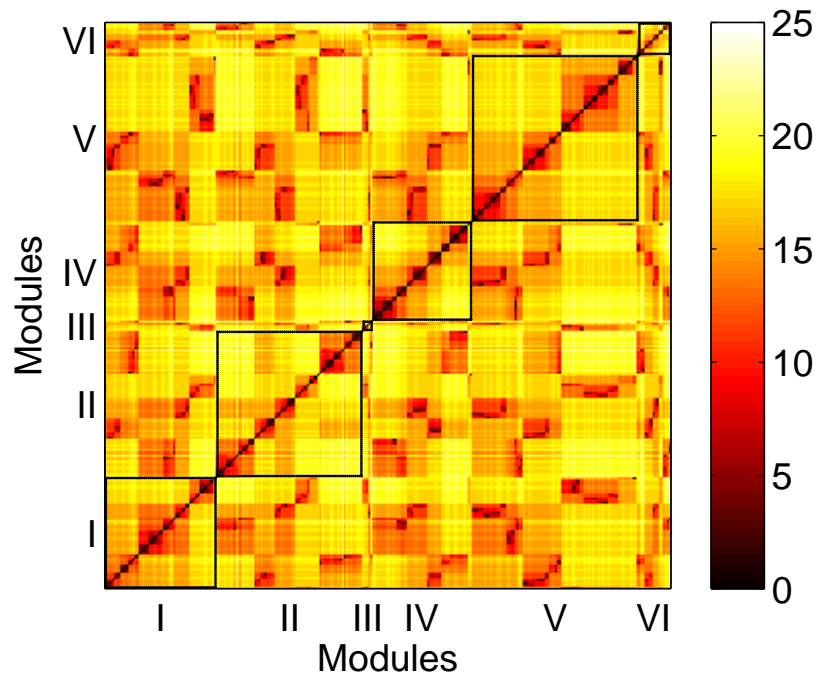

Supplement: Figure S1 — Matrix representing the relatedness of neurons in the somatic nervous system of C. elegans as measured in terms of their lineage distance. The neurons are arranged according to the modules they belong to. The module boundaries are indicated in the figure. Within each module, neurons that are close in terms of lineage are placed in adjacent positions. The figure shows that closely related neurons may occur in different modules, while those in the same module may be far apart in terms of lineage distance. This indicates that there is no simple relation between relatedness of neurons in terms of lineage and their modular membership. (0.05 MB PDF) [file pone.0009240.s001.pdf]

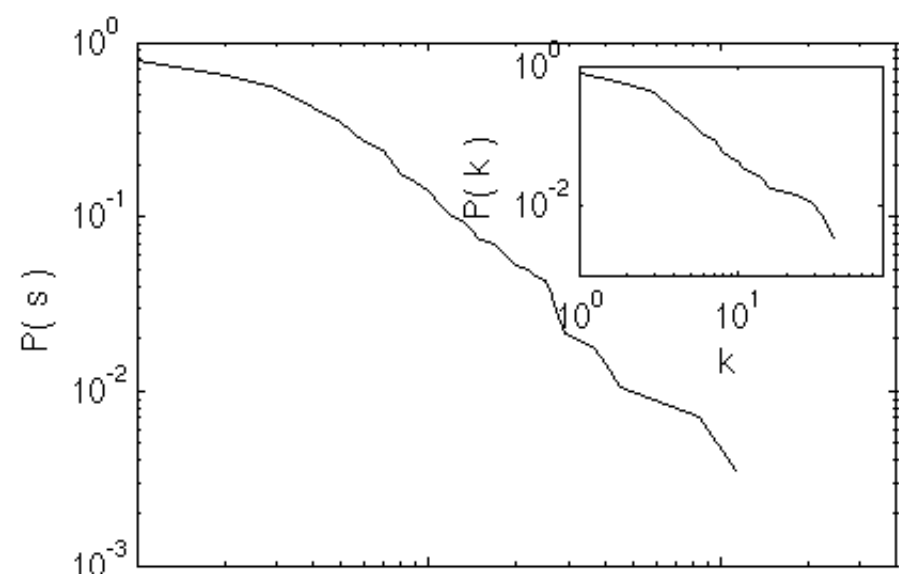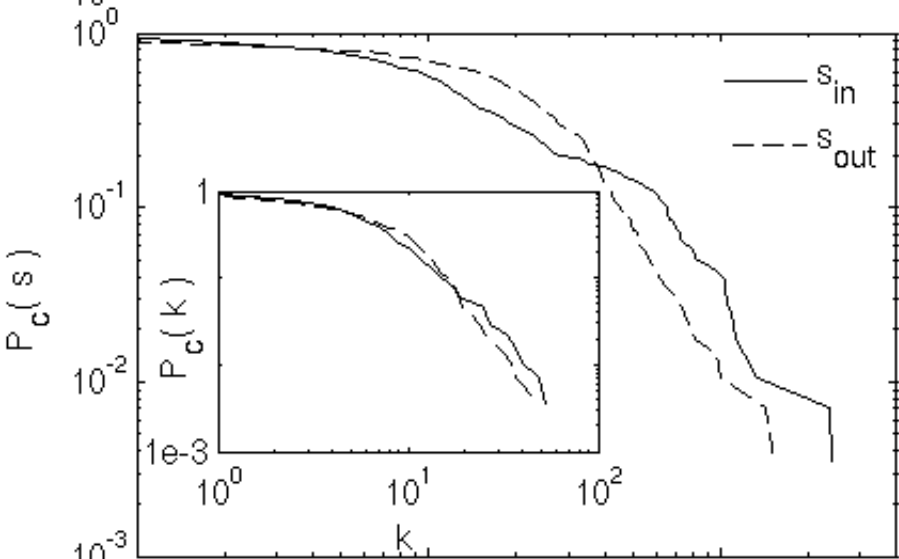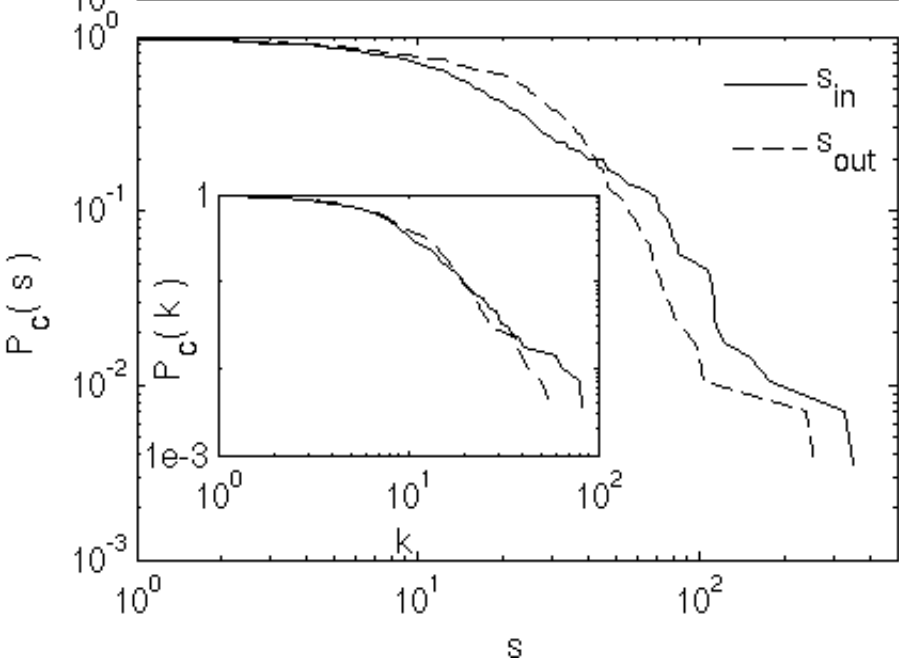

Supplement: Figure S2 — Cumulative distributions of the strength and (inset) degree for the (top) gap-junctional, (center) synaptic and (bottom) combined networks. The gap-junctional network is undirected and the strength of a node is defined as si = Σj Wij, where Wij is the number of gap junctions between neurons i and j. On the other hand, the synaptic and combined networks are directed and the inward- and outward-strength of a node are defined as si in = Σj Wji, and si out = Σj Wij, respectively. For directed networks, Wij represents the number of connections from neuron j to i. The figures indicate that scale-free behavior of the distributions is seen only for the gap-junctional network. The other two networks exhibit exponentially decaying nature for both the degree and the strength distributions. (0.01 MB PDF) [file pone.0009240.s002.pdf]

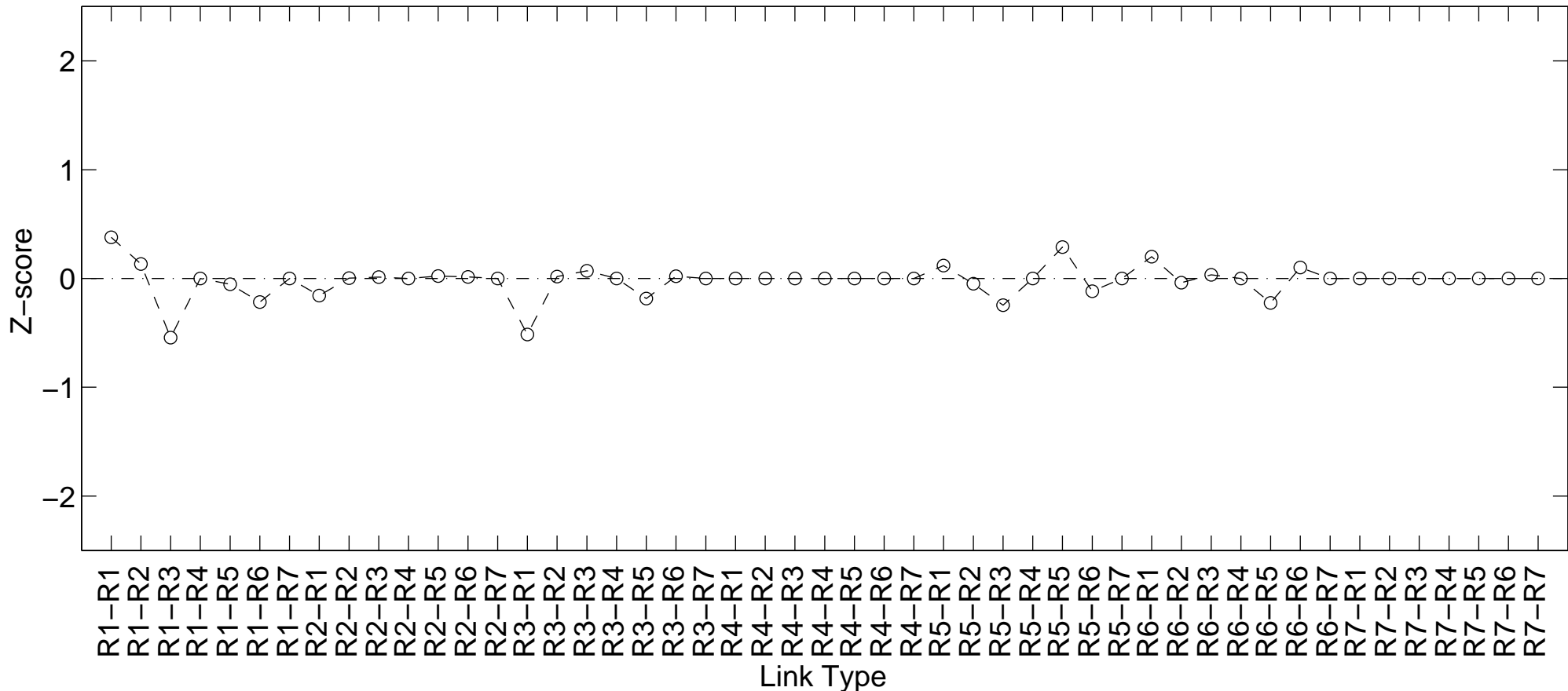

Supplement: Figure S4 — The role-to-role connectivity pattern indicated by the z-scores for abundance of links between each pair of roles (R1–R7) in C. elegans neuronal network. Note that, as there are no neurons having roles R4 or R7 in the empirical network, links from other roles to these two do not exist. The z-scores represent the abundance of links between each pair of roles in the C. elegans somatic nervous system with respect to degree- and modularity-preserved randomized ensemble of networks (103 realizations). The method used for calculating the z-score is as described in R. Guimera, M. Sales-Pardo and L.A.N. Amaral, “Classes of complex networks defined by role-to-role connectivity profiles”, Nature Physics, 3 (2007) 63–69. (0.01 MB PDF) [file pone.0009240.s004.pdf]
