# Supplementary material for: Mesoscopic Organization Reveals the Constraints Governing Caenorhabditis elegans Nervous System
Source: PLoS One. 2010 Feb 22;5(2):e9240. doi: 10.1371/journal.pone.0009240 (PMC2825259; doi:10.1371/journal.pone.0009240)
Supplement: Text S1 — Analysis of the C. elegans network of synaptic connections. (0.16 MB PDF) [file pone.0009240.s009.pdf]

Text S1 : Analysis of the C. elegans network of synaptic connections.

The analysis of the C. elegans nervous system in our paper done for the combined synaptic-gap junctional network has been repeated for the network of exclusively synaptic connections to verify the robustness of our results. The optimal partition of the synaptic network yields 4 modules. As in the analysis of the combined network reported in the paper, we observe the correlation of the synaptic network modules with neuronal interconnectivity, neuron type and individual cell body position. We also consider the overlap of these modules with the different ganglia and functional circuits. Finally, we look at the betweenness centrality and average nearest neighbor degree as a function of average neuron degree, as well as, the role of each neuron in terms of its intra- and inter-modular connectivity. The results of our analysis with the network of exclusively synaptic connections is qualitatively identical to that reported in the paper for the combined network, underlining their robustness with respect to how the network is defined.

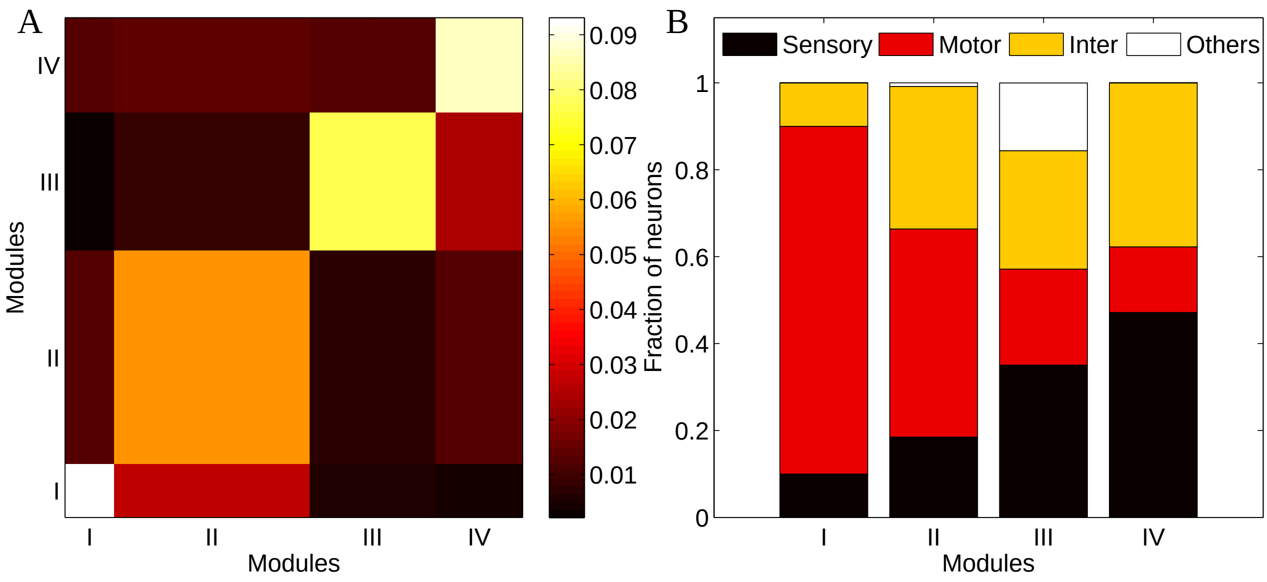

Figure 1: **Modular interconnectivity and the decomposition of modules of the synaptic network according to neuron type.** (A) Matrix representing the average connection density between neurons occurring within modules and those in different modules of the synaptic network. (B) The modules of the synaptic network are decomposed according to the different neuron types comprising them. The results are similar to that reported for the combined synaptic-gap junctional network.

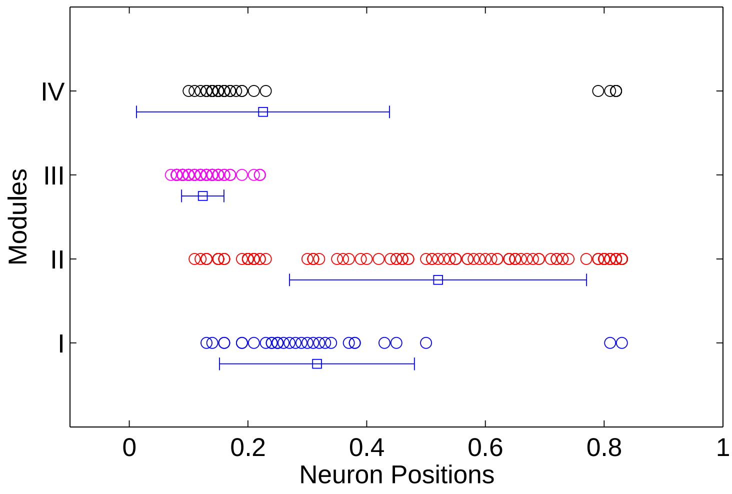

Figure 2: **Neuronal layout of the worm indicating cell body positions of each neuron.** The position of neuronal cell bodies along the longitudinal axis of the C. elegans body plan is shown, with the vertical offset and color indicating the module to which a neuron belongs. The optimal partitions for obtaining the modules are calculated using only the network of synaptic connections.

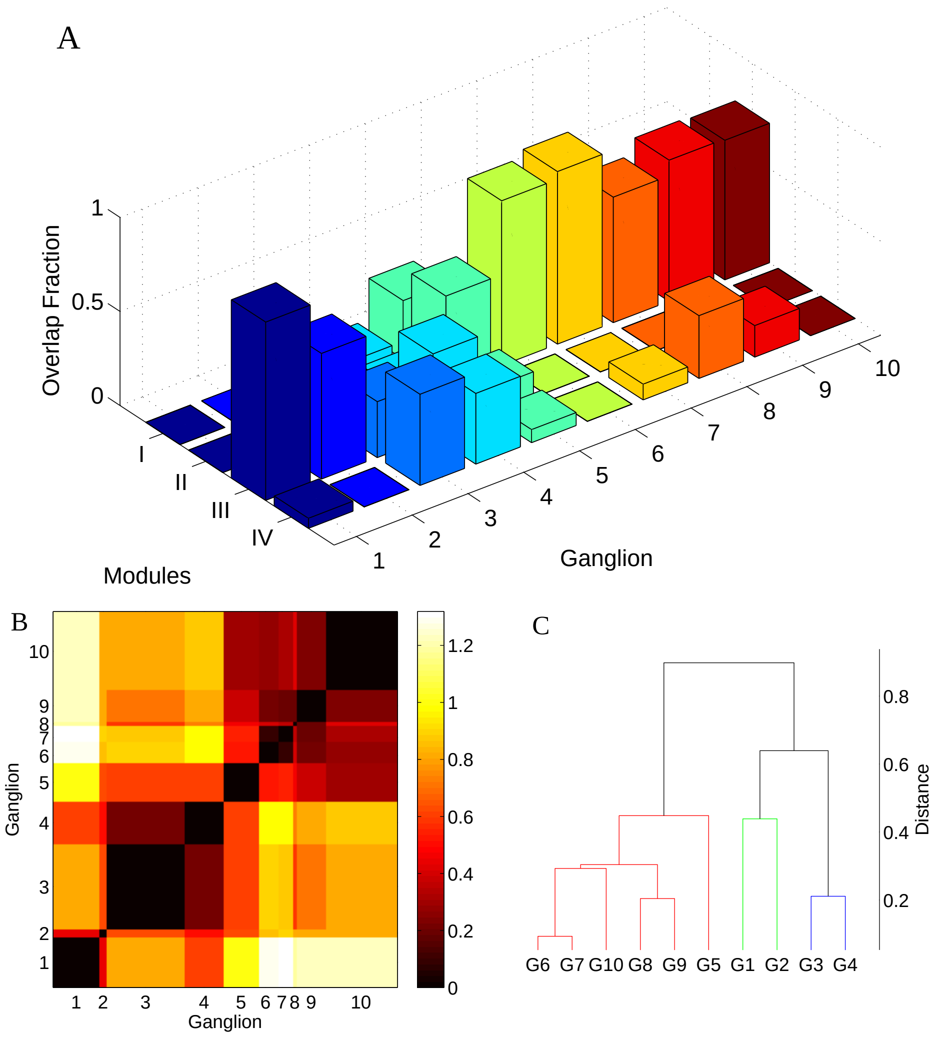

Figure 3: **Modular decomposition of different ganglia, with the modules obtained by optimal partitioning of the synaptic network.** (A) Neurons belonging to different ganglia are decomposed according to their modular membership. The height of each bar in the histogram corresponds to the overlap between the ganglia and the modules, calculated as the fraction of neurons that are common to a particular ganglion and a specific module. (B) The matrix representing the average modular distance between the different ganglia, as calculated from the modular decomposition spectrum of each ganglion. The corresponding dendrogram indicates the closeness between different ganglia in the abstract 4-dimensional "modular" space. (C) The matrix of physical distances between the ganglia is shown for comparison with (B), calculated as the average distance between neurons belonging to the different ganglia. The corresponding dendrogram indicated the closeness between ganglia according to the geographical nearness of their constituent neurons in the nematode body.

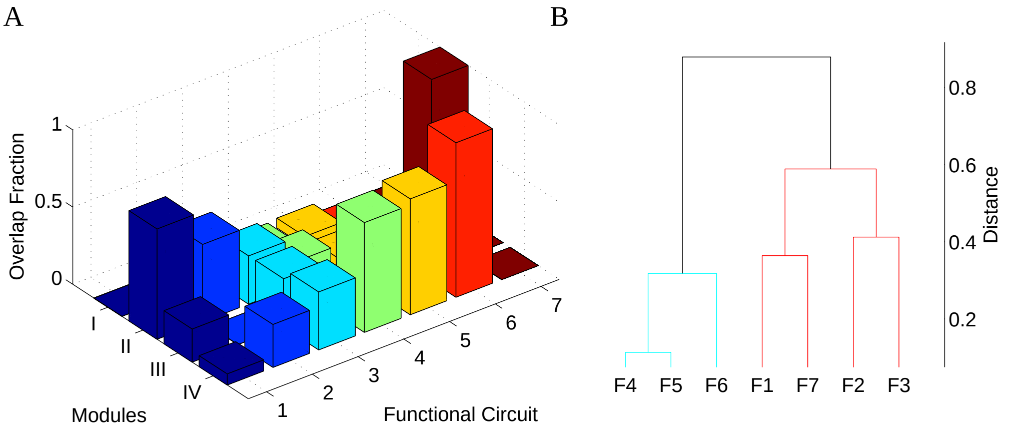

Figure 4: **Modular decomposition of neurons in different functional circuits, with the modules obtained by optimal partitioning of the synaptic network.** Neurons belonging to different functional circuits are decomposed according to their modular membership. The height of each bar in the histogram corresponds to the overlap between the modules and functional circuits (F1) mechanosensation, (F2) egg laying, (F3) thermotaxis, (F4) chemosensation, (F5) feeding, (F6) exploration and (F7) tap withdrawal. The overlap is measured in terms of the fraction of neurons common to a particular functional circuit and a specific module. The corresponding dendrogram represents the closeness between different functional circuits in the abstract 4-dimensional "modular" space.

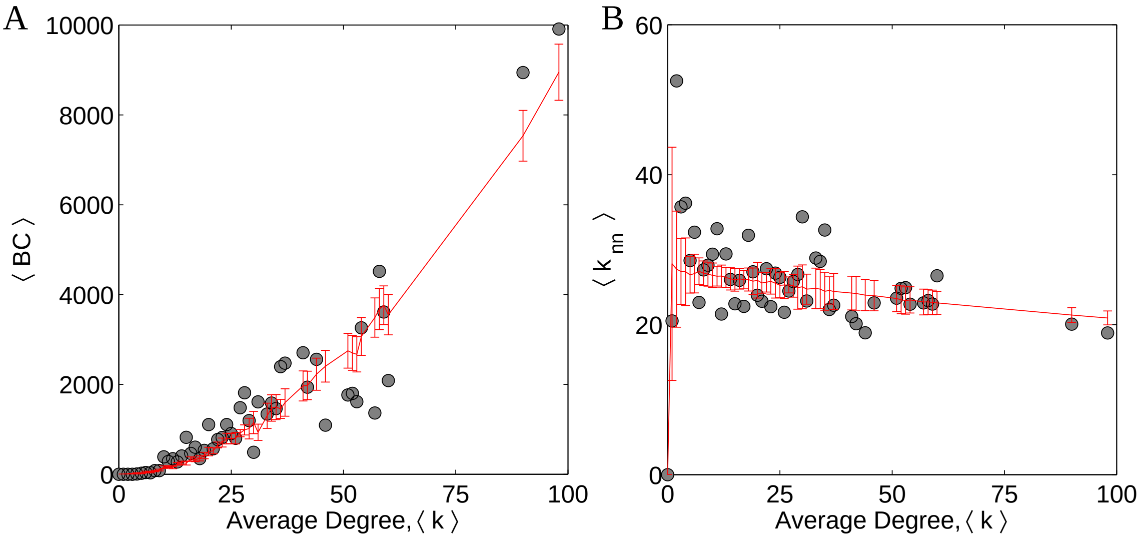

Figure 5: **Betweenness centrality and the average nearest neighbor degree as a function of the total degree of neurons in the synaptic network.** (A) The average betweenness centrality, $\langle BC\rangle$, and (B) the average nearest neighbor degree, $\langle k_{nn}\rangle$ of each node as a function of its total degree, $\langle k\rangle = \langle k_{\rm in} + k_{\rm out}\rangle$. In both figures, a comparison is made with the randomized version of the network and the error bars indicate the standard deviations calculated for 10^3 random realizations.

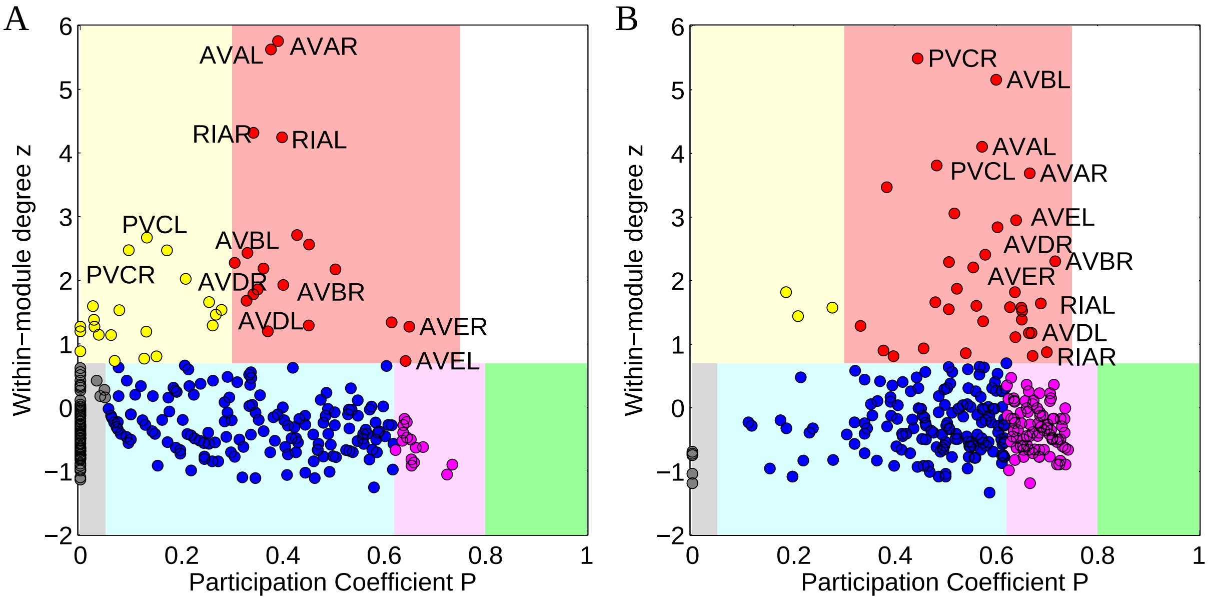

Figure 6: **The role of individual neurons according to their intra- and inter-modular synaptic connectivity.** (A) The within-community degree z-score of each neuron in the empirical neuronal network is plotted against the corresponding participation coefficient P. The corresponding result for a randomized version of the C. elegans network where the degree of each neuron is kept unchanged is shown in (B).
