# Supplementary material for: Mesoscopic Organization Reveals the Constraints Governing Caenorhabditis elegans Nervous System
Source: PLoS One. 2010 Feb 22;5(2):e9240. doi: 10.1371/journal.pone.0009240 (PMC2825259; doi:10.1371/journal.pone.0009240)
Supplement: Figure S3 — The intra- and inter-modular connectivity of individual neurons in the C. elegans somatic nervous systems, color-coded to represent the different ganglia in which each occurs. (Top) The within module degree z-score of each neuron in the empirical neuronal network is shown against the corresponding participation coefficient P. (Bottom) The corresponding result for a randomized version of the C. elegans network where the degree of each neuron is kept unchanged. The lateral ganglion is seen to occupy a prominent position in the system, coordinating the information flow between the neuronal groups responsible for receiving sensory stimuli and those controlling motor activity. On the other hand, the randomized network shows similar prominence for several other ganglia. (0.04 MB PDF) [file pone.0009240.s003.pdf]

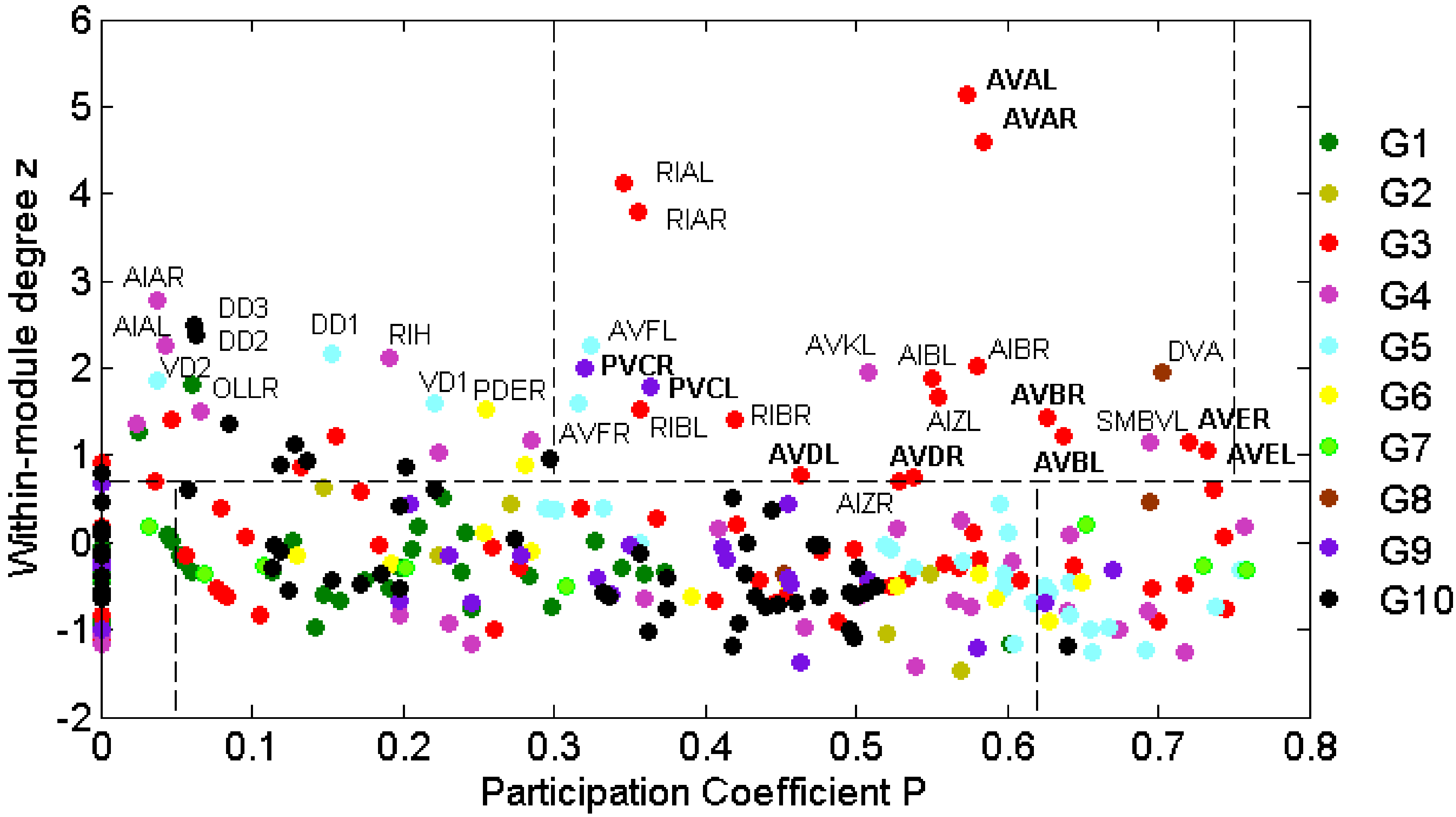
Within-module degree z
Participation Coefficient P
6
5
4
3
2
1
0
-1
-2
0
0.1
0.2
0.3
0.4
0.5
0.6
0.7
0.8
AVAL
AVAR
RIAL
RIAR
AIAR
AIAL
DD3
DD2
VD2
OLLR
DD1
RIH
VD1
PDER
AVFL
PVCR
PVCL
AVFR
RIBL
RIBR
AVKL
AIBL
AIBR
DVA
AVBR
AIZL
SMBVL
AVER
AVEL
AVDL
AVDR
AVBL
AIZR
G1
G2
G3
G4
G5
G6
G7
G8
G9
G10

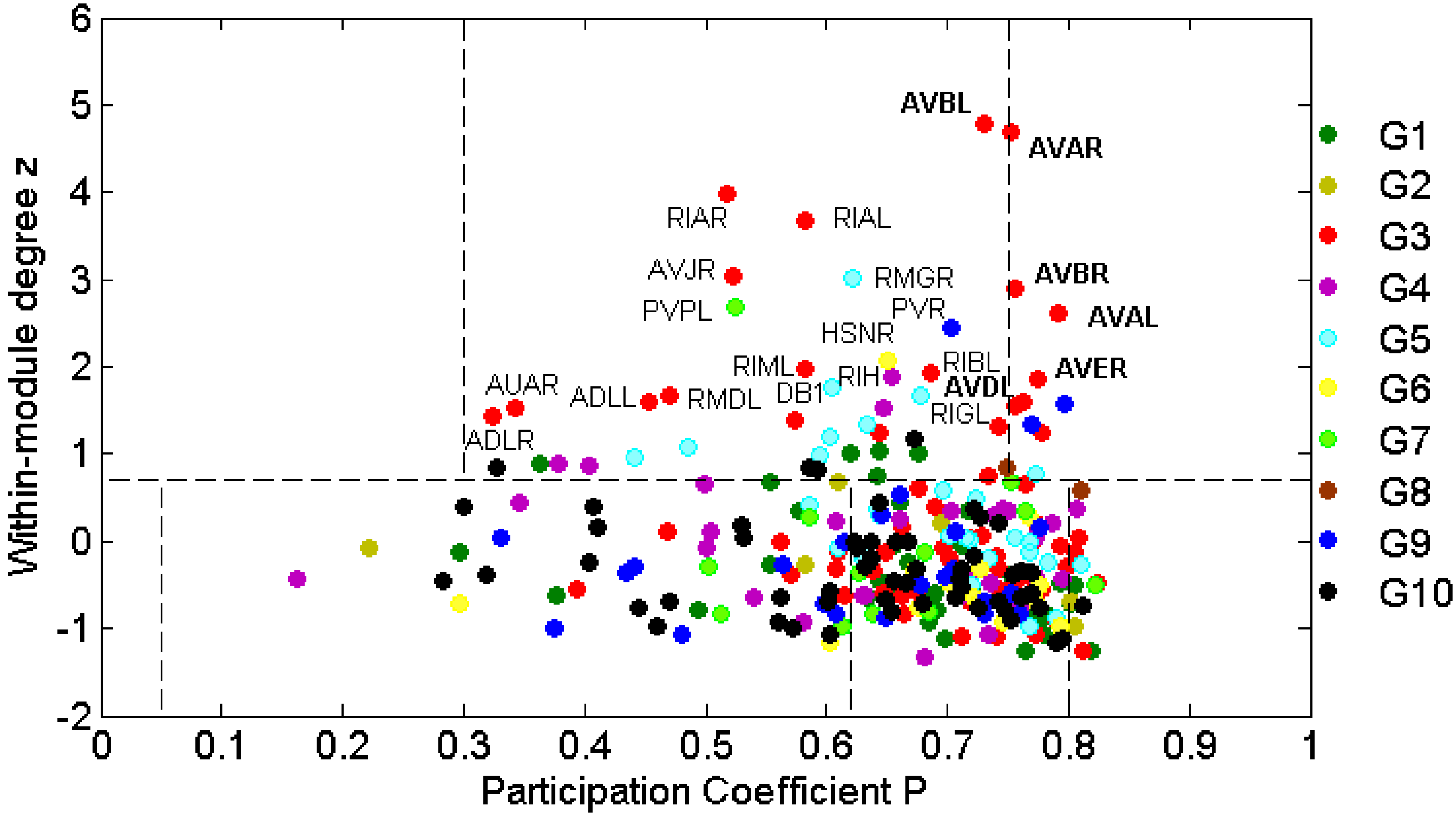
Within-module degree z
Participation Coefficient P
6
5
4
3
2
1
0
-1
-2
0
0.1
0.2
0.3
0.4
0.5
0.6
0.7
0.8
0.9
1
AVBL
AVAR
RIAR
RIAL
AVJR
RMGR
AVBR
PVPL
PVR
AVAL
HSNR
RIML
RIBL
RIH
AVER
AUAR
ADLL
RMDL
DB1
AVDL
RIGL
ADLR
G1
G2
G3
G4
G5
G6
G7
G8
G9
G10
